# Extracellular Vesicles From 3xTg-AD Mouse and Alzheimer’s Disease Patient Astrocytes Impair Neuroglial and Vascular Components

**DOI:** 10.3389/fnagi.2021.593927

**Published:** 2021-02-19

**Authors:** Luis Alfonso González-Molina, Juan Villar-Vesga, Julián Henao-Restrepo, Andrés Villegas, Francisco Lopera, Gloria Patricia Cardona-Gómez, Rafael Posada-Duque

**Affiliations:** ^1^Área de Neurobiología Celular y Molecular, Grupo de Neurociencias de Antioquia, Universidad de Antioquia, Medellin, Colombia; ^2^Facultad de Ciencias Exactas y Naturales, Instituto de Biología, Universidad de Antioquia, Medellin, Colombia; ^3^Neurobank, Neuroscience Group of Antioquia, Faculty of Medicine, SIU, University of Antioquia, Medellin, Colombia

**Keywords:** 3xTg-AD mice, astrocytes, human astrocytes, extracellular vesicles, endothelial disruption, familiar Alzheimer’s disease, sporadic Alzheimer disease

## Abstract

Astrocytes are specialized glial cells that are essential components of the neurovascular unit (NVU) and are involved in neurodevelopment, brain maintenance and repair, and neurodegeneration. Astrocytes mediate these processes by releasing cellular mediators such as extracellular vesicles (EVs). EVs are vehicles of cell-cell communication and have been proposed as mediators of damage in AD. However, the transcellular mechanism by which Alzheimer disease (AD) astrocytes impair the function of NVU components is poorly understood. Therefore, we evaluated the effects of adult PS1-KI and 3xTg-AD astrocyte conditioned media (CM) and EVs on NVU components (neuroglia and endothelium) *in vitro*. Additionally, SAD and FAD astrocyte-derived EVs (A-EVs) were characterized, and we evaluated their effects on NVU in cocultured cells *in vitro* and on intrahippocampal CA1 cells *in vivo*. Surprisingly, cultured 3xTg-AD astrocytes showed increased glial fibrillary acidic protein (GFAP) reactivity compared to PS1-KI astrocytes, which denotes astrocytic hyperreactivity. CM from adult mice 3xTg-AD astrocytes increased cell-cell gaps between endothelial cells, filopodia-like dendritic protrusions in neurons and neuronal and endothelial cell death. 3xTg-AD A-EVs induced neurotoxicity and increased astrocyte GFAP reactivity. Cultured human *postmortem* astrocytes from AD patients also increased GFAP reactivity and EVs release. No differences in the size or number of A-EVs were detected between AD and control samples; however, both SAD and FAD A-EVs showed increased expression of the surface marker aquaporin 4. A-EVs induced cytotoxicity and astrocyte hyperactivation: specifically, FAD A-EVs induced neuroglial cytotoxicity and increased gaps between the endothelium, while SAD A-EVs mainly altered the endothelium. Similarly, both AD A-EVs increased astrocyte GS reactivity and vascular deterioration *in vivo*. We associated this finding with perivascular reactive astrocytes and vascular deterioration in the human AD brain. In summary, these results suggest that AD A-EVs impair neuroglial and vascular components.

## Introduction

The neurovascular unit (NVU) is a functional structure composed of the endothelial microvasculature that makes up the blood brain barrier (BBB), neurons, astrocytes, pericytes, microglia and the basal lamina (Hawkins and Davis, 2005; Muoio et al., 2014). The NVU controls blood flow through the BBB, regulating the immune response and providing trophic support to the brain to maintain cerebral homeostasis and the microenvironment (Lok et al., 2007; Muoio et al., 2014). In neurodegenerative processes, the structure and function of NVU cells are affected, leading to loss of the repair capacity of the brain parenchyma and neuronal connections and increased excitotoxicity and apoptosis (Guo and Lo, 2009; Iadecola, 2017). Thus, each cell type that makes up the NVU is crucial for its proper functioning, and functional alterations in only one cell type can induce damage in the other cell types, exacerbating the damage and inducing the development of diseases (Girouard et al., 2012; Attems and Jellinger, 2014).

Astrocytes are crucial for neurodevelopment and the maintenance and repair of the cellular microenvironment in the NVU (Rothstein et al., 1996; Aubert et al., 2007; Attwell et al., 2010; Mishra et al., 2016; Boulay et al., 2017; Tapella et al., 2018). During development, astrocytes are involved in synaptogenesis and synaptic plasticity (Banker, 1980; Allen and Eroglu, 2017; Diniz et al., 2017; Ruffinatti et al., 2018). Astrocytes convey chemical signals between neighboring neurons and modulate the exchange of neuroactive factors (Ghézali et al., 2016); additionally, astrocytes project their “endfeet” toward endothelial cells to cover the vascular system of the CNS (Mathiisen et al., 2010) and regulate the peripheral immune system and metabolic blood supply in response to neuronal activity (Abbott et al., 2010a; Alvarez et al., 2013; Boulay et al., 2016). Dysfunctional astrocytes are involved in CNS diseases, including AD (Rothstein et al., 1996; Sofroniew and Vinters, 2010; Olabarria et al., 2011; Grolla et al., 2013; Stenovec et al., 2016; Ruffinatti et al., 2018). Astrocytes respond to pathological conditions through the process of astrogliosis (Sofroniew and Vinters, 2010; Haim and Rowitch, 2016), which is characterized by increased reactivity and morphological and metabolic changes that trigger the uptake and elimination of toxic substances (Iram et al., 2016; Diniz et al., 2017). However, maintenance of astrocytes in a reactive state results in the loss of their normal functionality and induces neuronal cell loss (Iram et al., 2016; Bronzuoli et al., 2018). Astrocytes release a broad variety of cellular mediators, such as cytokines, growth factors, neurotransmitters, and extracellular vesicles (EVs) (Farina et al., 2007; Schitine et al., 2015; Verkhratsky et al., 2016).

Extracellular vesicles are heterogeneous cell-derived membranous vesicles that differ in size and biogenesis and include exosomes (50–150 nm), microvesicles (100–1000 nm), and apoptotic bodies (up to 5 μm). EVs are considered common vehicles of intercellular communication in homeostatic and pathological processes. EVs can transfer nucleic acids, lipids, proteins (functional enzymes) and organelles (mitochondria) (Yáñez-Mó et al., 2015; Van Niel et al., 2018). In particular, EVs can promote the spreading of protein aggregates (Pérez et al., 2019). EVs are increased in AD patient cerebrospinal fluid, and these EVs contain the functional protease calpain (Laske et al., 2015) and induce neuronal death (Joshi et al., 2013). A focus of EV studies in AD has been EVs from the brain parenchyma; microglial EVs have been found to deliver toxic forms of amyloid β and IL-1β and induce neuronal death (Yang et al., 2018). Astrocyte-derived EVs (A-EVs) regulate brain homeostasis by providing bioenergetic support to neurons (Hayakawa et al., 2016). In a neuroinflammatory context, A-EVs can mediate leukocyte infiltration (Dickens et al., 2017), have a higher expression of complement (De Luca et al., 2018; Goetzl et al., 2018), and alter neuronal branching and firing (You et al., 2020). Therefore, we propose that astrocyte EVs could be mediators of neurodegenerative diseases such as AD.

Alzheimer disease is the major cause of elderly dementia (Querfurth and Laferla, 2010). Genetic early-onset familial AD (FAD) is associated with autosomal dominant mutations in APP (amyloid precursor protein), PS1 (presenilin-1) or PS2 (presenilin-2). While most AD cases are sporadic late-onset AD (LOAD), 1–2% of cases are familial early-onset AD (EOAD) with underlying mutations in presenilin-1 and presenilin-2 (PSEN1/2) (Waring and Rosenberg, 2008). Notably, the largest and most homogeneous family group reported to date with familial Alzheimer’s disease, known as the family group with the “paisa” E280A mutation of the Presenilin (PSEN) gene, is located in Antioquia (Lopera et al., 2013). Previous work by our group has identified a differential lipid profile in the brain (Villamil-Ortiz et al., 2018) and differential EVs in the circulation of SAD and FAD patients that is related to NVU degeneration (Villar-Vesga et al., 2020).

3xTg-AD is a transgenic mouse model used in AD research that develops plaques of accumulated Aβ (6 months) and hyperphosphorylated tau (12 months) as they age (Oddo et al., 2003a), thus mimicking the onset and progression of AD (Oddo et al., 2003b). 3xTg-AD astrocytes exhibit dysfunctional Aβ plaque uptake and impaired neuroprotective function that are associated with low expression levels of C1q-associated scavenger B1 receptor (Iram et al., 2016). Similarly, early atrophy of 3xTg-AD astrocytes induces the widening of synaptic clefts. These effects may generate defective synaptic modulation and cognitive impairment in AD (Yeh et al., 2011). Purified cultures of 3xTg-AD hippocampal astrocytes exhibit transcriptional alterations (Ruffinatti et al., 2018). Additionally, immortalized hippocampal astrocytes from 3xTg-AD mice had an impaired ability to support BBB integrity *in vitro* through paracrine mechanisms (Kriaučiūnaitë et al., 2020). It has been recently shown that astrocytes from 3xTg-AD mice exhibit impaired vesicle trafficking and mobility *in vitro* (Stenovec et al., 2016). Thus, studying the effect of EVs released by 3xTg-AD astrocytes on the cellular components of the NVU can contribute to knowledge of the etiopathology of AD.

Similarly, human *in vitro* models have been developed to elucidate the role of astrocytes in AD (Guttenplan and Liddelow, 2019). These models include iPSC-derived astrocytes, cerebral organoids and postmortem astrocyte cultures (Zhang et al., 2016; Sloan et al., 2017; Guttenplan and Liddelow, 2019). Thus, postmortem astrocyte cultures could preserve the microenvironmental context of AD in humans and be a useful tool for studying astrocytes (Liddelow and Barres, 2017; Spaethling et al., 2017). In *in vitro* AD models, astrocyte function has been associated with disease pathology hallmarks (González-Reyes et al., 2017), such as protein aggregates, altered lipid trafficking (González-Reyes et al., 2017), altered cellular trafficking and an altered secretome (Stenovec et al., 2016). We propose EVs as conveyers of astrocyte alterations in human AD.

Therefore, our study aimed to evaluate the effect of A-EVs from 3xTg-AD mice and human SAD and FAD on NVU components (astrocytes, neurons and endothelium) *in vitro* and *in vivo* to provide essential information for understanding the role of astrocytes in the pathophysiology of AD.

## Materials and Methods

### Animals

All animal procedures were performed in accordance with the ARRIVE guidelines, the Guide for the Care and Use of Laboratory Animals (8th edition) published by the National Institutes of Health (NIH) and Colombian standards (law 84/1989 and resolution 8430/1993). These procedures were approved by the Ethics Committee for Animal Experimentation at the University of Antioquia, Medellin, Colombia.

### Primary Neuronal Cultures

Cortical neurons were extracted from rat embryos at 15 days of gestation (*n* = 3). The neurons were enzymatically dissociated using a 0.25% trypsin/EDTA mixture (Gibco^TM^, 15400054) and seeded in 24-well plates (Falcon^®^) on poly-L-lysine-pretreated coverslips (Sigma-Aldrich, P2636) in Dulbecco’s Modified Eagle Medium (DMEM) (Sigma-Aldrich, D5648) containing 10% horse serum (HS) (Gibco^TM^, 16050122). 3 h after seeding, the neurons were plated at a density of 52 cells/mm^2^, and DMEM was switched to Neurobasal^TM^ medium (Gibco^TM^, 21103049) supplemented with B-27^®^ (Gibco^TM^, 17504044) and 0.25% L-glutamine (L-Gln) (Sigma-Aldrich). All media contained 1% penicillin-streptomycin (Gibco^TM^, 15140122). At DIV3, the neurons were treated with AraC (Sigma-Aldrich) to inhibit the proliferation of non-neuronal cells and were incubated at 37°C in an atmosphere of 5% CO_2_ for up to 19 days *in vitro* (DIV19) (Posada-Duque et al., 2015).

### Endothelial Cell Line Culture

The mouse brain microvasculature cell line bEnd.3 (ATCC CRL-2299) was used as an endothelial cell model. bEnd.3 cells were thawed in DMEM supplemented with 20% fetal bovine serum (FBS) (Gibco^TM^, 16000044) and a 1% mixture of penicillin-streptomycin. After 24 h, the thawing medium was switched to maintenance medium (DMEM supplemented with 10% FBS and 1% penicillin-streptomycin mixture). The cells were incubated at 37°C in 5% CO_2_, subcultured using a 0.25% trypsin/EDTA mixture for 5 min and seeded in 12- or 24-well plates at densities of 2.5 × 10^4^ or 1.5 × 10^4^ cells per coverslip (Becerra-Calixto et al., 2018).

### Primary Culture of Astrocytes From Adult and Neonatal Mice

Depending on the experimental design, primary astrocyte cultures were purified from the brain cortex of adult (8–9 months; C57B/6, *n* = 4; PS1-KI, *n* = 7; 3xTg-AD, *n* = 8) or newborn (postnatal day 1 or 2, *n* = 3) mice. Cortical tissues were enzymatically dissociated with a 0.25% trypsin/EDTA mixture, cultured in T75 (surface area 75 cm^2^, for neonate cells) or T25 (surface area 25 cm^2^, for adult cells) flasks, incubated at 37°C in 5% CO_2_ and maintained in DMEM supplemented with 10% FBS and a 1% penicillin-streptomycin mixture. The culture medium was changed every 2 days. The adult astrocytes were confluent on DIV27, and subsequently, the culture media and all soluble release factors were collected to perform the corresponding treatments (Posada-Duque et al., 2015; Becerra-Calixto et al., 2018). Neonatal astrocytes (DIV8) in the flasks were placed in a shaker at 37°C at 350 rpm for 6, 18, and 24 h. During these time intervals, each culture flask was washed with 1 × phosphate-buffered saline (PBS), and the culture medium was renewed to remove oligodendrocytes and microglia. The cells reached confluence on DIV10. Subsequently, neonatal astrocytes were subcultured using a 0.25% trypsin/EDTA mixture for 15 min and seeded in 12-well plates at a density of 7.5 × 10^4^ cells per coverslip.

### Primary Rat Astrocyte Cultures

Cortical samples from newborn Wistar rats on postnatal days 1–2 were enzymatically dissociated with trypsin, cultured in T75 (surface area 75 cm^2^) flasks at 37°C and 5% CO_2_, and maintained in DMEM supplemented with 10% FBS. The medium was changed every 2 days. The neonatal astrocytes (DIV8) in the flasks were placed in a shaker at 37°C at 350 rpm for 6, 18, and 24 h. During these time intervals, each culture flask was washed with 1 × PBS, and the culture medium was renewed to remove oligodendrocytes and microglia. Cells reached confluence on DIV10. Subsequently, neonatal astrocytes were subcultured using a 0.25% trypsin/EDTA mixture for 15 min and seeded in 12-well plates at a density of 7.5 × 10^4^ cells per coverslip (Posada-Duque et al., 2015; Becerra-Calixto et al., 2018).

### Coculture of Endothelial and Neonatal C57BL6, PS1-KI, and 3xTg-AD Astrocytes

Cocultures were performed and modified according to a previous experimental design (Becerra-Calixto et al., 2018). bEnd.3 cells were thawed and subcultured to DIV4 in 12-well plates. The cells were cultured on gelatin-pretreated coverslips with paraffin dots (size of dots: approximately 0.5 mm height and 2 mm width). In parallel, primary astrocytes were subcultured on coverslips in 12-well plates at a density of 7.5 × 10^4^ cells per coverslip. bEnd.3 cells and primary DIV10 astrocytes were used for coculture. After the astrocytes reached DIV29, the cocultures of the two cell types were fixed for cellular analyses.

### Treatments With CM

(i) Endothelial CM

**FIGURE 1:**
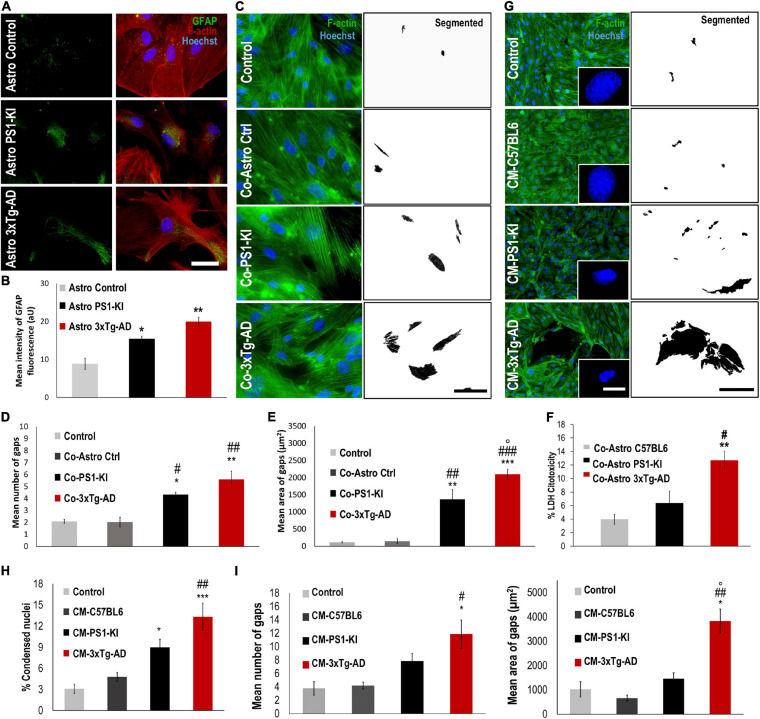
CM from 3xtg-AD astrocytes induced endothelial alterations in the context of AD. **(A)** DIV 23-25 culture of control, PS1-KI and 3xTg-AD astrocytes. Reactivity of 3xTg-AD astrocytes. Hoechst staining of the nuclei is blue. Anti-GFAP-Alexa 488 staining of GFAP is green. Phalloidin-Alexa 594 staining of F-actin is red Calibration bar: 50 μm. **(B)** GFAP fluorescence intensity quantification of 3xTg-AD astrocytes. Significant increase in GFAP intensity in treated 3xTg-AD astrocytes compared to that in the control samples. *n* = 3. Comparison with *, Control astrocytes Glu-; #, Control Coculture Glu-. *P-value* significance: **0.01; *0.05. **(C)** Endothelial cells cocultured with astrocytes from neonate 3xTg-AD. Hoechst staining of the nuclei is blue. Phalloidin-Alexa 594 staining of F-actin is green. Threshold details of cell-cell spaces in coculture with PS1-KI and 3xTg-AD astrocytes. Scale bar: 50 μm. **(D)** Coculture with 3xTgAD astrocytes induced significant changes in the number of endothelial cell gaps. Ten fields per treatment were analyzed for each n. **(E)** Coculture with 3xTgAD astrocytes induced an increase in the average total area of gaps *in vitro*. Ten fields per treatment were analyzed for each n. *n* = 3. Comparison with *Control; #Co-Astro ctrl; °Co-Astro PS1-KI. *P-value* significance: *** and ^###^0.001; ** and ^##^0.01; *, ^#^, and °0.05. **(F)** Coculture with 3xTg-AD astrocytes increased cytotoxicity in endothelial cells. *n* = 3. Comparison with *Co-Astro C57BL6; #Co-Astro PS1-KI. *P-value* significance: **0.01; ^#^0.05. **(G)** Endothelial cells treated with CM from astrocytes for 24 h. Hoechst staining of the nuclei is blue. Phalloidin-Alexa 488 staining of F-actin is green. Details of condensed nuclei magnified to 100%; scale bar 4 μm. **(H)** Average percentage quantification of condensed nuclei. Comparison with *Control; #CM-C57BL6; °CM-PS1-KI. *P-value* significance: ***0.001; ^##^0.01; *0.05. **(I)** Treatment with CM from 3xTgAD astrocytes induced a significant increase in the number and area of gaps between endothelial cells cultured *in vitro*. Twenty-five fields per treatment were analyzed for each *n*. *n* from 4 to 8; comparison with *Control; #CM-C57BL6; °CM-PS1-KI. *P-value* significance: ^##^0.001; *, ^#^, and °0.05.

When primary astrocyte cultures from adult mice reached 70% confluence (Figure 1A), CM was aspirated and used to treat endothelial cells previously seeded in 24-well plates. Each well was treated with 300 μL of CM. The endothelial cells were maintained for 24 h in the CM treatment medium, and the cells were fixed with 4% paraformaldehyde prepared in 1 × cytoskeleton buffer with sucrose (CBS).

(ii) Neuron CM

When primary astrocyte cultures from adult mice reached 70% confluence (Figure 1A), the culture medium was changed to Neurobasal^TM^ medium supplemented with B-27 and a mixture of penicillin-streptomycin antibiotics. On the next day, CM from these cultures was aspirated and added to neurons (DIV15) seeded in 24-well plates. Each well was treated with 300 μL of CM. The neurons were maintained for 24 h in the presence of CM and were fixed for cellular analyses.

### Mouse EVs Analysis

Culture media from C57BL6, PS1-KI and 3xTg-AD astrocytes were collected for EV isolation. A-EVs were isolated and processed from 1 mL CM as described previously (Sellam et al., 2009; Nielsen, 2012; Cloutier et al., 2013). CM was centrifuged twice at 3,000 *g* for 20 min at 21°C to remove apoptotic bodies and cell debris. Then, the supernatants were centrifuged at 16,900 *g* for 60 min at 21°C to obtain the EVs. The upper 800 μL corresponding to the soluble fraction was recovered, and the remaining 200 μL was the EV fraction, which was brought to a final volume of 1 mL with filtered commercial 1X PBS (Gibco, Thermo Fisher Scientific). To determine the size and number of EVs, the size parameters of the samples were evaluated (forward scatter, FSC-A) using a flow cytometer calibrated with polystyrene spheres (Polysciences, Inc.) of known sizes (0.5, 1, and 2 μm). Filtered PBS was used as a reference to determine the threshold of the measurements for all samples. Commercial spheres of known concentration (Beckman Coulter, IM3630E) were used to determine the EVs concentration in 1 mL of the sample. The EV pellets were immediately frozen in 100 μL of filtered Dulbecco’s phosphate-buffered saline (DPBS 1X, Gibco BRL) at −70°C until use for characterization or cell stimulation. The EV number corresponded to the number of total events registered during the assay after correction for the dilution factor and the read time. All samples were finally washed with 1X filtered PBS and immediately processed by a Beckman Coulter CytoFLEX flow cytometer (to measure the events with sizes of 100 nm, 160 nm, 200 nm and 300 nm) and a BD LSRFortessa instrument (Becton Dickinson, San Jose, CA) (to measure the events with sizes of 0.1, 0.5, 1, and 2 μm) using the FACS DIVA program provided by the manufacturer. Data analysis was performed with FlowJo 7.6.1 software (Tree Star, Inc., Ashland, OR, United States).

### Purification and Culture of Human Astrocytes

Briefly, we dissected meninges and blood spots from cortical brain tissue, incubated the tissue in trypsin 1x DNase for 15 min, and washed the tissue with astrocyte plating medium [containing DMEM (Gibco) with 10% fetal bovine serum (FBS, Gibco), 1X L-glutamine 0.25% (Sigma-Aldrich), 1X penicillin/streptomycin, and 1X antimycotic (Gibco)]. Then, we gently triturated the tissue 10–15 times and centrifuged it at 2000 rpm for 4 min. We resuspended the cell suspension and plated it in T25 culture flasks. After 24 h, we changed half of the medium, and after 48 h, we changed all medium to astrocyte medium (containing half DMEM, half Neurobasal (Gibco), 10% FBS, 1X N2, Epithelial Growth Factor (EGF, Sigma-Aldrich), 1X L-glutamine, bovine serum albumin (BSA), 1X penicillin/streptomycin, and 1X antimycotic) (Zhang et al., 2016). We kept the cells in astrocyte medium, changing the medium every 2–3 days, and observed cell proliferation after 2–3 weeks. Additionally, as an extra CNT, we thawed H9 hESC-derived NSCs (Gibco, catalog N7800-100, N7800-200) at passage 5 and cultured them according to the manufacturer’s instructions. After 2 weeks of culture, we changed the medium to astrocyte medium.

### Isolation of Human Postmortem A-EVs

At DIV 50 for human primary astrocytes, at DIV 15 for mouse primary astrocytes and at DIV 14 for NSCs, we started collecting half of the medium every 3 days. We pooled together media from 6 different medium change dates for a total of 2 weeks. From these samples, we purified A-EVs by differential centrifugation. Briefly, we centrifuged the medium at 3000 *g* for 20 min to remove apoptotic bodies and cell debris. Then, we centrifuged the medium at 17000 *g* for 1 h and resuspended the A-EV pellet in fresh PBS for further experiments.

### Phenotype of A-EVs

Human and mouse A-EV concentrations and sizes were evaluated as previously described for plasma EVs (Villar-Vesga et al., 2020). Briefly, we acquired all events present in 100 μL of cell medium. Using reference polystyrene spheres (0.1, 0.5, 1, 2, 3, and 6 μm, Polysciences), we compared EV distribution in FSCA parameters (size). A-EVs were characterized as previously described for plasma-EVs (Burbano et al., 2018; Villar-Vesga et al., 2019) by staining with anti-human astrocyte AQ4 FITC (Polyclonal, Bioss) (Nekludov et al., 2017) antibodies, detecting phosphatidylserine (PS) with Annexin V PE (Abcam), and detecting mitochondria using a DIOC6 (dihexyloxacarbocyanine iodide) probe (Invitrogen); we acquired samples in LSR Fortessa (BD). The percentage of EVs positive for each fluorochrome was determined using the fluorescence minus one (FMO) method. The mean fluorescence intensity (MFI) was determined for both negative and positive EVs. We acquired the data in LSR Fortessa (BD), the number of EVs was analyzed, and all analyses were performed using FlowJo 7.6.1 software (Burbano et al., 2018).

### Coculture of Neurons and Astrocytes From Rats

To obtain primary neurons, cortical samples from Wistar rat embryos (E18–19) were dissected and dissociated, and 1 × 10^5^ neurons were cultured on 18-mm coverslips with paraffin “feet” (paraffin dots adhered to the coverslips, approximately 0.5 mm high and 2 mm wide) that were precoated with polylysine (Posada-Duque et al., 2015). The cells were cultured in neurobasal medium (Gibco) with 1X B-27 supplement (Sigma-Aldrich), 0.25% L-glutamine and 1X penicillin-streptomycin antibiotic mixture (Gibco) at 37°C in a 5% CO_2_ humidified atmosphere. At DIV10 for the neurons, the coverslips containing the neurons were moved to the astrocyte plates, suspended on the astrocytes, and kept in serum-free neurobasal medium for 7 days. At DIV17 for the neurons, the cocultures were treated with A-EVs for 24 h.

### Coculture of bEnd.3 Cells and Rat Astrocytes

bEnd.3 cells were subcultured on previously gelatinized coverslips and paraffin dots (approximately 0.5 mm high and 2 mm wide). In parallel, primary rat astrocytes were subcultured in 12-well plates (Becerra-Calixto et al., 2018). On DIV16, the bEnd.3 cells and primary rat astrocytes were cocultured for 4 days. On DIV 20 for astrocytes, the cocultures were stimulated with A-EVs for 24 h.

### Preparation and Stimulation With A-EVs

We stimulated all the above cell cultures with C57BL6, PS1-KI and 3xTg-AD A-EVs or CNT, SAD, and FAD A-EVs at a cell:EV ratio of 1:1. The EVs were thawed at room temperature, washed with 1X PBS, and centrifuged at 16900 × *g* for 1 h; then, predetermined amounts of EVs were added at a 1:1 ratio. The cell cultures were stimulated for 24 h with EVs resuspended in fresh medium. As a control, we added fresh medium without EVs. Mouse A-EVs were used to stimulate neuron and astrocyte cocultures. Human A-EVs were used to stimulate neuron-astrocyte and astrocyte-endothelium cocultures. For 10-month-old mice, EV treatment for 48 h was chosen according to the approximate number of cells in CA1 and following our previously published protocol using organoids (Villar-Vesga et al., 2020). All stimulations were performed in medium depleted of EVs by filtration through a 0.1-μM membrane.

### Cell Culture Immunofluorescence

Cell cultures were fixed with 4% paraformaldehyde prepared in 1 × CBS (Posada-Duque et al., 2017). Autofluorescence was eliminated using 50 mM NH_4_Cl. The cells were permeabilized with 0.2% Triton X-100 prepared in 1 × CBS and subsequently blocked with blocking solution (2.5% FBS in 1 × CBS). The culture plates were incubated overnight at 4°C with primary mouse antibodies against MAP2 (1:750, Sigma-Aldrich, 2-G3893) to stain neurons, GFAP (1:750, Sigma-Aldrich, 3-M4403), s100β (Dako, IS504) and GS (1:500, Molecular Probes; 701989) to stain astrocytes, and IBA-1 (1:500, Wako, 019-19741) to stain microglia, and p120 ctn (1:1000, Sigma-Aldrich, 15D2) to stain endothelium. Subsequently, the samples were incubated with secondary Alexa 594 or 488 antibodies (1:500, Molecular Probes), Hoechst 33258 (1:5,000, Invitrogen) and phalloidin conjugated to Alexa 488 or 594 (1:500, Molecular Probes). The coverslips were mounted onto slides with FluorSave (Calbiochem). Observation and image capture of cells were performed using an Olympus IX 81 epifluorescence microscope with 20 × objective (NA, 0.5) and 60 × oil immersion objective (NA 1.42) lenses. The dendritic spines in neurons were imaged using an Olympus IX 81 DSU spinning disc confocal microscope with a 60 × objective lens (NA, 1.42) with immersion oil.

### Morphometric Cell Culture Analysis

(i) Quantification of gaps between endothelial cells

To determine the number of “gaps” between endothelial cells, Image-Pro Plus and Adobe Photoshop software were used. In Adobe Photoshop, a binary image of the gaps in each captured field of observation (at 20×, 25 fields for each n; at 60×, 10 fields for each n) was generated by carefully tracing each gap with the “magic wand” tool. From this binary image, the number and area of the gaps were measured using the “Count and measure objects” tool of Image-Pro Plus software.

(ii) Quantification of F-actin spots in endothelial cells

The number and area of F-actin spots in endothelial cells were measured using FIJI-NIH software. The images were segmented with the “Trainable Weka Segmentation” plugin (Arganda-Carreras et al., 2017). Three categories of segmentation were used: background, actin cytoskeleton fibers and F-actin spots. Binary images of the F-actin spots were generated in each captured field of observation (at 60×, 25 fields).

(iii) Quantification of the number and length of dendritic spines

The spines were counted using deconvoluted images (Xcellence, Olympus) converted to 8-bit images. Excess background illumination was subtracted from a value of 25 using the Image-Pro Plus Subtract tool. Finally, the spines protruding from 10-μm proximal dendrites (primary dendrites) from the soma were manually quantified using the Image-Pro Plus “Count and measure objects” tool.

(iv) Quantification of the percentage of condensed nuclei

The percentage of condensed nuclei was calculated using the following equation: % of condensed nuclei = [condensed nuclei/(condensed nuclei + normal nuclei)] ^∗^ 100. The area, perimeter and diameter of each nucleus were quantified in the Hoechst staining images (20 ×) using the Image-Pro Plus “Count and measure objects” tool. Nuclei with diameters from 3.0 to 4.5 μm were considered condensed in the case of neurons, and nuclei with diameters from 3.0 to 6.0 μm were considered condensed in the case of endothelium.

(v) Quantification of GFAP in astrocytes

The mean intensity of GFAP fluorescence was quantified in GFAP-positive images (60×) using the FIJI-NIH “Count and measure objects” tool.

(vi) Analysis of astrocyte/endothelium and astrocyte/neuron cocultures

The sample size was defined as the number of patients (n) or mice (n). All the measurements are reported as fold changes. The fold change data were calculated by dividing the values obtained from cells after treatment with CNT, SAD and FAD A-EVs or C57BL6, PS1-KI and 3xTg-AD A-EVs by the value obtained from cells with no EV treatment. In astrocytes from both coculture conditions, we measured the mean fluorescence intensity (MFI) of GFAP per field (at 20×, 12 fields per n), and in the endothelium, we measured the MFI of p120 catenin per field (at 20×, 12 fields per n). The MFI was obtained by measuring the mean gray value of each field. In addition, we measured sizes of gaps in the endothelium (Becerra-Calixto et al., 2018). For this measurement, we binarized F-actin images using the same threshold for all conditions. The mask area was considered as the gaps. Within the mask area, we calculated gap size using particle size analysis (at 20×, 12 fields per n). We used a similar strategy in the neuron images. Additionally, we measured the fold change in the percentage of condensed nuclei in the neuron images. For this measurement, we binarized Hoechst images and defined condensed nuclei as nuclei with an average area less than 40 μm^2^ (at 20×, 12 fields per n).

For human astrocytes, we measured the MFI of GFAP as described above. We measured the mean gray value in five fields per *n* at 20×. Then, we averaged the mean gray value for each n and reported it in relative units. Additionally, we normalized the MFI of GFAP from SAD and FAD human astrocytes to that from CNT astrocytes. All analysis was performed in ImageJ Software.

### Lactate Dehydrogenase (LDH) Release Cytotoxicity Assay

Cytotoxicity associated with the coculture of endothelium and 3xTg-AD, PSI-KI and C57BL6 astrocytes and in response to human- or mouse-derived A-EVs was measured. The activity of LDH released from the cells due to membrane disruption was measured using a Roche Cytotoxicity Detection Kit (LDH). On coculture DIV 17 (DIV 27 for the astrocytes and DIV 21 for the endothelial cells), the culture medium was collected, and LDH activity was determined as a linear rate of NADH consumption during the reduction of pyruvate to lactate using a spectrophotometer. The percentage of cytotoxicity was calculated for each treatment using the following equation:%Cytotoxicity = [(A- low control)/(high control- low control)]^∗^100, where A is the average of three replicates of LDH activity measurements for each treatment, low control is LDH activity released from the untreated cells (spontaneously released LDH), and high control is the maximum LDH activity released from the cells (treated with 1% Triton X-100 for 24 h). The colorimetric signal was read at 490 nm using a BioRad ELISA reader (Roche).

### *In vivo* Injection of A-EVs

A-EVs pools isolated from control (*n* = 3), SAD (*n* = 4), and FAD (*n* = 5) astrocyte media were quantified and resuspended at 20 × 10^3^ EVs/μL. C57BL6 mice were anesthetized using ketamine (90 mg/kg) and xylazine (5 mg/kg) and received a 2–4% isoflurane and 96% oxygen mixture via an inhalation anesthesia machine during injection. Each mouse (*n* = 3) was injected bilaterally with CNT A-EVs in the right hemisphere and FAD or SAD A-EVs in the left hippocampus using the following coordinates: bregma: −1.94 mm, lateral: ±0.8 mm, depth: −1.5 mm. A total of 40 × 10^3^ EVs were resuspended in 2 μL of filtered Dulbecco’s phosphate-buffered saline (1X DPBS, GibcoBRL) and placed in a syringe for subsequent injection. The injections were performed via a delivery pump with a Hamilton syringe 26 s/2″/2 (Hamilton^®^ Reno, NV, United States) to a maximum volume of 2 μL at a rate of 0.15 μL/min; a 5-min wait was implemented before withdrawal of the syringe. Postinjection, the animals were kept alive for 48 h before sacrifice.

### Human Samples and Tissues

We included *postmortem* samples from 3 CNT cases and 1 astrocyte culture derived from neural stem cells (NSCs). We included *postmortem* brain samples from *n* = 6 CNTs, *n* = 4 SADs (LOAD), *n* = 6 FADs (E280A) and *n* = 3 FADs (samples from donors carrying the E280A mutation and from those with a familial inheritance pattern of AD having more than 3 relatives in the same generation with the disease were considered as FAD, respectively) from the University of Antioquia Biobank. The following sample data are included in Table 1: sex, age of onset, age of death, *postmortem* index (PI) as the time elapsed between the patient’s death and sampling, Consortium to Establish a Registry for Alzheimer’s Disease (CERAD), Braak staging to classify the degree of AD, Thal phase as an intersection of tau and Aβ, NIA-AA criteria as a pathological diagnosis of AD high likelihood, and comorbidities. We obtained approximately 2–4 cm^3^ of frontal lobe cortical tissue from each patient for astrocyte cultures and histological analyses. Tissue for astrocyte culture was immersed in 1 × 4°C PBS and transferred to the lab for tissue dissociation immediately after resection; tissue for histological analyses was immersed in 37°C PFA 4% prepared in cytoskeleton buffer.

**TABLE 1:** Description of post-mortem samples.

Condition	Sex	Age of onset	Age of death	P.I. (h)	CERAD	Braak	Thal	NIA-AA	Comorbilities
Control	M	NAP	69	6.8	A	1	2	A1, B1, C1	Hypertension, Chronic venous insufficiency
Control	M	NAP	61	5.3	0	0	0	A0, B0, C0	Hypertension, type II diabetes mellitus
Control	F	NAP	44	4.4	0	0	0	A0, B0, C0	Depression
Control	M	NAP	38	3.4	0	0	0	A0, B0, C0	None
Control	M	NAP	73	3.5	B	1	2	A1, B1, C2	None
Control	M	NAP	46	6	0	1	0	A0, B1, C0	None
Control	M	NAP	84	3	0	3	0	A0, B2, C0	Hypertension, Chronic renal insufficiency, Chronic obstructive lung disease
FAD (E280A)	F	44	50	5.2	C	6	5	A3, B3, C3	None
FAD (E280A)	M	46	62	4.3	C	6	5	A3, B3, C3	Hypertension, alcoholism, Traumatic brain injury
FAD (E280A)	M	49	59	5	C	6	5	A3, B3, C3	Dyslipidemia, alcoholism
FAD (E280A)	F	41	56	5.0	C	5	5	A3, B3, C3	Hypertension
FAD (E280A)	M	44	57	1.8	C	5	5	A3, B3, C3	Traumatic brain injury
FAD (E280A)	M	42	53	2.8	C	6	5	A3, B3, C3	None
FAD	F	82	90	3.2	C	3	3	A2, B2, C3	Hypothyroidism, Chronic venour insufficiency, COPD
FAD	F	74	86	3.3	B	5	4	A3, B3, C2	Hypothyroidism, Dyslipidemia
FAD	M	68	73	6.3	C	5	3	A2, B3, C3	None
SAD	F	81	94	4	B	4	5	A3, B2, C2	Hypertension, type II diabetes mellitus, breast cancer
SAD	F	79	90	3,3	C	4	4	A3, B2, C3	Hypertension, Dyslipidemia
SAD	F	59	73	3.5	C	5	4	A3, B3, C3	Dyslipidemia, Depression
SAD	F	92	98	4.3	A	3	3	A2, B2, C1	Hypertension

*Clinical data of patients used for the different experimental procedures. 0, no histological evidence of Alzheimer’s disease; A, histological findings provide UNCERTAIN evidence of Alzheimer’s disease; B, histological findings SUGGEST the diagnosis of Alzheimer’s disease; C, histologic findings indicate the diagnosis of Alzheimer’s disease. F, female; M, male; NAP, not applicable; P.I., Postmortem Index.*

### Immunofluorescence of Human and Mouse Brain Samples

We obtained middle frontal gyrus cortex samples with very low *postmortem* delays (mean of 4.87 h) from CNT, SAD and FAD donors. Immediately after collection, the samples were fixed with 4% paraformaldehyde (PFA) prepared in cytoskeleton buffer for 72 h at 4°C, and the solution was renewed every 24 h. Alternatively, anesthetized mice were perfused with cardiac puncture using normal saline solution (0.9% NaCl) and then PFA. The perfused brains were extracted and fixed as described for the human brain samples. Subsequently, the samples were sectioned into coronal slices of 50 μm thickness with a Leica cryostat. Before immunostaining, antigen retrieval was performed in the human slices using 98% formic acid at 85°C for 5 minutes. Autofluorescence was blocked using 50 mM NH4Cl. To avoid non-specific binding of the antibodies, the samples were incubated in 1% bovine serum albumin (BSA, Sigma-Aldrich, A9647) for 1 h at room temperature (RT). The brain slices were incubated for 72 h at 4°C in the primary antibodies mouse CLN-5 (Invitrogen; 35-2500; 1:500) and rabbit GFAP (Invitrogen; PA5-16291; 1:500) diluted in antibody solution containing 0.3% BSA, 0.3% Triton-X100 and 1 M phosphate buffer (PB; pH 7.4). After the excess antibodies were removed by 20 minutes of washing, the slices were incubated for 1 h at RT in secondary antibody solution with goat anti-mouse Alexa Fluor 488 (Invitrogen; A-11001; 1:750) and goat anti-rabbit Alexa Fluor 594 (Invitrogen; A-11012; 1:750); the endothelial marker DyLight 649 lectin UEA I (Vector Labs; DL-1068; 1:500) was also incubated with the human slices. Later, the samples were vigorously washed in 1 M PB 3 times for 5 min each. Finally, the slices were mounted on glass slides with FluorSave Reagent (Millipore; 345789).

### Confocal Microscopy of Human and Mouse Brain Samples

Microvessels in the gray matter were captured by confocal microscopy. Three high-magnification images per slide were obtained from triple immunolabeling. In the case of human samples, the images were acquired using a Perkin Elmer Ultraview RS Spinning Disk Confocal Microscope equipped with a 12−bit CCD camera (Hamamatsu ORCA-ER), a 40X oil-immersion objective (NA 1.3; C−Apochromat; Zeiss), two diode lasers of 488 and 655 nm (Omicron) and Volocity 4.2 software (Improvision). The images were obtained using a 1.5-second exposure time and an optobar of 1.6X. Sixteen-bit TIFF images of 1344 × 1024 pixels (139.44 × 106.24 μm) in size were taken with an XY pixel size of 103.75 nm and a distance of 300 nm between Z−sections. Approximately 50 optical slices (15 μm thick) of each field were captured. Mouse samples stained for GS-CLN-5 were captured using an Olympus FV1000 confocal scanning microscope equipped with a 60X oil-immersion objective (NA 1.42; PLAPON; Olympus) with a zoom value of 2, three lasers of 405, 488, and 543 nm and FluoView 3.1.1.9 software (Olympus). Sixteen-bit TIFF images of 1024 × 1024 pixels (105.47 × 105.47 μm) were obtained with an XY pixel size of 103 nm and a distance of 300 nm between Z−sections. Thirty optical slices of each field were captured (9 μm thick).

### Confocal Image Processing and Analysis of Human Brain Samples

Confocal images were deconvolved, processed, and segmented, and 3D reconstructions were created. The images were deconvolved in Huygens Professional 19.10 software (Scientific Volume Imaging B.V.). Images of human samples were deconvolved using the classic maximum likelihood estimation (CMLE) algorithm with a signal-to-noise ratio of 40, and those from EVs injection in mice were deconvolved using the deconvolution express algorithm. The images were transformed to 8 bits and subsequently processed and analyzed in FIJI software. Immunofluorescence signals were segmented using intensity thresholding by the Otsu algorithm to standardize the fluorescence signals from all the images. The total and colocalizing stained areas were measured. The colocalized signals were obtained using the AND algorithm from the image calculator tool. The 3D Distance Map tool from the 3D Suite plugin (Ollion et al., 2013) was used to characterize the association of astrocytic processes with the vasculature, excluding signals coming from 2 μm away from the vessel surface. Z projections by standard deviation of the segmented stacks were used to evaluate vascular structure. Thus, we analyzed the intensity of the superposed UEA I signal coming from different optical slices as a measurement of the thickness of the vascular wall. These Z projections were segmented and used to quantify the vessel gaps. The 3D ROI Manager from the 3D Suite plugin (Ollion et al., 2013) was employed to determine the surface area of the vessels. Last, for illustrative purposes, Z projections of the deconvolved images were made using the max intensity option.

### Statistical Analysis

For the data acquired, we tested normality using the Shapiro–Wilk and Kolmogorov normality tests. If data were normally distributed, parametric analysis was used. ANOVA tests were used to compare the mouse samples; comparisons of independent groups (CM in neurons: *n* = 3; CM in endothelium: *n* = 4-8; GFAP astrocytes of newborn mice *n* = 3; coculture of endothelium and astrocytes: *n* = 3) were performed using the Tukey-Kramer *post hoc* test. Kruskal-Wallis and *post hoc* Dunn’s tests at α = 0.05 were used to assess the significance level of the results obtained using the data that did not meet the assumption of normality. For human samples, the sample size for each experimental group was defined as each patient and analyzed as an independent assay. Parametric univariable data were analyzed using one-way ANOVA followed by Tukey’s multiple comparison test. Non-parametric data were analyzed using the Kruskal–Wallis test and Dunn’s multiple comparison test. For multivariable analysis, two-way ANOVA was used followed by Bonferroni *post hoc* test for comparisons between several independent groups. Since we did not intervene in our human experimental groups but instead described differences between natural diseased populations, pairwise comparisons were also performed with unpaired two-tailed Student’s t-test when both data sets were normally distributed. We processed all groups in parallel to reduce the interassay variation. The data are expressed as the means plus the SEM or medians plus interquartile ranges depending on the normality test. The results were considered significant at *P-*values below the significance level (α = 0.05^∗^, 0.010^∗∗^, or 0.001^∗∗∗^).

## Results

### Hyperreactive 3xTg-AD Astrocytes Induced Endothelial Gaps

Glial fibrillary acidic protein reactivity is a hallmark of astrocytes reacting to pathological conditions; reactive astrocytes induce phenotypic changes that allow them to recognize and eliminate potentially toxic substances (Eng et al., 2000). Therefore, we assessed GFAP reactivity in primary astrocyte cultures from C57BL6 (control), PS1-KI and 3xTg-AD mice. The purity of the astrocyte cell cultures was verified with different labeling methods (Supplementary Figure 1). PS1-KI and 3xTg-AD astrocytes constitutively showed higher GFAP expression than control astrocytes (Figures 1A,B). Thus, the results suggest that PS1-KI and 3xTg-AD astrocytes were hyperreactive. To determine the effect of neonatal 3xTg-AD astrocytes on endothelial cells, the number and area of the gaps between endothelial cells cocultured with each astrocyte type were quantified (Figure 1C). Endothelial cells cocultured with PS1-KI and 3xTg-AD astrocytes showed increased numbers and areas of gaps compared with the control cells (Figures 1D,E). Coculture of endothelial cells with 3xTg-AD astrocytes showed significantly increased cytotoxicity compared with cocultures of endothelial cells with C57BL6 or PSI-KI astrocytes (Figure 1F). Therefore, we evaluated the effect of CM from adult PS1-KI and 3xTg-AD astrocytes (Figure 1G). Endothelial cells treated with PS1-KI astrocyte CM showed an increase in condensed nuclei; however, this effect was stronger in cells treated with 3xTg-AD astrocyte CM (Figures 1G,H). Interestingly, endothelial cells treated with any type of CM showed consistent F-actin spots (Supplementary Figure 2A); specifically, KI-PS1 and 3xTg-AD astrocyte CM increased the number and area of these F-actin spots in endothelial cells (Supplementary Figure 2B). These results suggest that adult PS1-KI and 3xTg-AD astrocytes reduced endothelial cell viability in association with an increase in F-actin aggregation. Remodeling of the actin cytoskeleton is related to the gaps between endothelial cells, which result in the loss of cell-cell junctions (Becerra-Calixto et al., 2018). Based on these considerations, we quantified the number and area of gaps formed between endothelial cells treated with astrocyte CM (Figure 1I) and found that adult 3xTg-AD astrocyte CM treatment induced increased numbers and areas of gaps between endothelial cells compared with the control conditions (Figure 1I). These findings suggest that 3xTg-AD astrocytes exhibit hyperreactivity and impair endothelial cells.

### Adult 3xTg-AD Astrocyte CM Induced Neurotoxicity and Filopodia-Like Dendritic Protrusions

**FIGURE 2:**
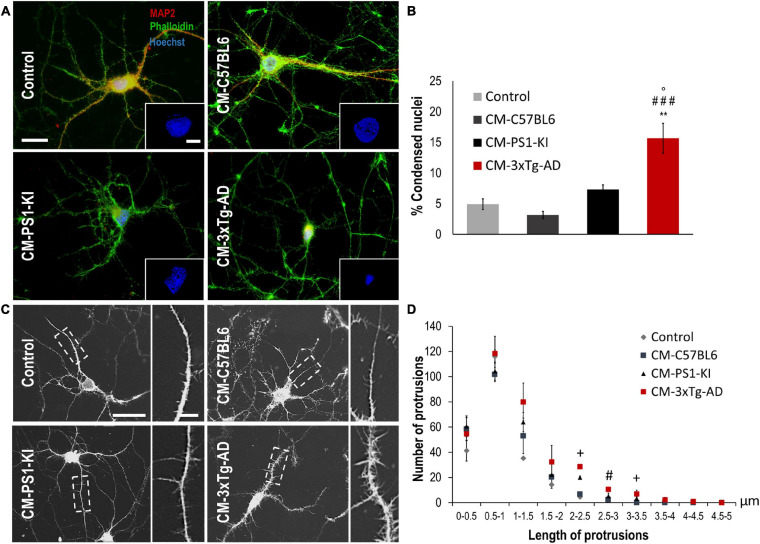
CM from adult 3xTg-AD astrocytes decreased viability and generated filopodia-like dendritic protrusions in neurons *in vitro*. **(A)** Immunofluorescence of neurons treated with CM from control or 3xTg-AD astrocytes. Hoechst nuclear staining is blue. Phalloidin-Alexa 488 staining of F-actin is green. MAP2-Alexa 594 staining of microtubule-associated protein 2 is red. Calibration bar: 20 μm. Details of condensed nuclei at 50% zoom, calibration bar: 5 μm. **(B)** Quantification of the average percentage of condensed nuclei. Average number of condensed nuclei was compared in various treatments. *n* = 3; ANOVA comparisons of 3xTg-AD with *Control; #CM-C57BL6; °CM-PS1-KI. *P-value* significance: ^###^0.001; **0.01; °0.05. **(C)** Neurons exposed to CM of astrocytes during 24 h; calibration bar: 20 μm. Details in the dotted rectangle show 100% magnification of the dendrites with the spines; calibration bar: 5 μm. **(D)** Distribution of the number of spines according to the length range. *n* = 3; ANOVA comparisons of 3xTg-AD with #CM-C57BL6. *P*-value significance: #0.05. Kruskal-Wallis analysis of non-normally distributed data. *P*-value significance: +0.05.

Astrocyte factors are essential for neuron viability and the development of dendritic spines (Murai et al., 2003; Procko and Shaham, 2010; Clarke and Barres, 2013). To assess whether adult PS1-KI or 3xTg-AD astrocytes influence neuronal viability, we treated DIV15 neurons with CM from each astrocyte type (C57BL6, PS1-KI and 3xTg-AD) (Figure 2A), quantified the percentage of condensed nuclei and evaluated neuron morphology. Neurons treated with adult 3xTg-AD astrocyte CM showed an increase in condensed nuclei (Figures 2A,B). CM from adult PS1-KI and 3xTg-AD astrocytes induced morphological changes in dendritic spines (Figure 2C). Treatment with adult PS1-KI astrocyte CM increased the ratio of filopodia-like spines in dendrites, but this effect was enhanced by CM from adult 3xTg-AD astrocytes (Figures 2C,D). Considering that filopodia-like dendritic protrusions are over 2.0 μm in length (Figure 2C), it should be noted that the CM from adult 3xTg-AD astrocytes induced a significant increase in the number of spines with lengths between 2.0 and 3.5 μm (Figure 2D). These results suggest that a factor released from adult 3xTg-AD astrocytes causes neuronal impairment.

### Adult 3xTg-AD Mice Have an Increase in A-EV Number

**FIGURE 3:**
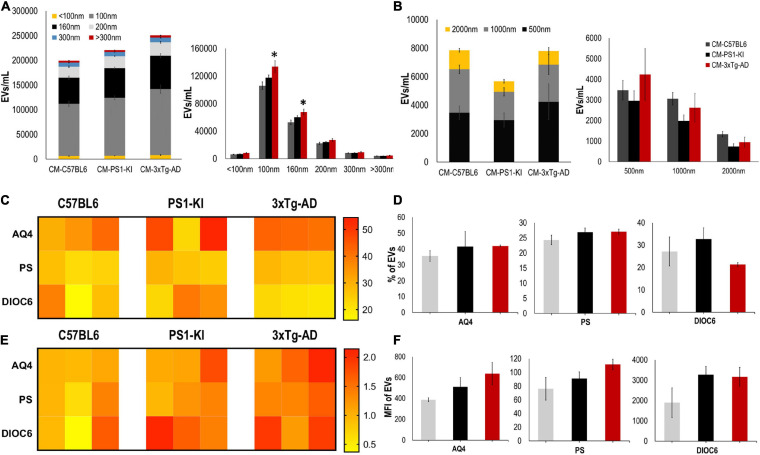
3xTg-AD mice have an increase in A-EVs. **(A)** Quantification and distribution of EVs numbers from CM treatment according to diameter (<100 and 100 nm: exosomes; 160, 200, and 300 nm: MVs). Significant differences in the number of 100 nm exosomes and 160 nm MVs in 3xTg-AD astrocytes compared with that in the C57BL6 samples. *P*-value significance *0.05. *n* from 4 to 8. **(B)** Quantification and distribution of EVs numbers from CM treatment according to diameter (500; 1000; and 2000 nm). No significant differences in the number of EVs in 3xTg-AD astrocytes compared with that in the other samples. *n* from 4 to 8. **(C)** Heatmap of percentages of EVs positive for cell markers (AQ4, astrocyte; Annexin, phosphatidyl-serine and DIOC6, mitochondria) from each astrocyte group. **(D)** Comparative of percentages of EVs positive of cell markers for grouped data seen in panel **(C)**. **(E)** Heatmap of the normalized MFI of cell markers (to mean data from C57BL6 individuals). **(F)** Comparative of MFI of cell markers for grouped data seen in panel **(E)**. Representative data from *n* = 3. Data were plotted as means and SEM. Kruskal–Wallis test and Dunn’s multiple comparison test. ^∗^Indicates *p* < 0.05; ^∗∗^ indicates *p* < 0.01.

Recently, it was shown that EV release is crucial for intercellular communication and viability (Frühbeis et al., 2013; Holm et al., 2018; Pascua-Maestro et al., 2019), and altered EV production may cause neurodegeneration (Basso et al., 2013; Frühbeis et al., 2013; Chaudhuri et al., 2018); thus, we measured and characterized the EVs found in CM. CM from adult 3xTg-AD astrocytes had a higher number of EVs than that from control astrocytes; specifically, we found a significant increase in the numbers of exosomes (100 nm) and MVs (160 nm) (Figure 3A) but not in the number of EVs with larger diameters (500, 1000, or 2000 nm) (Figure 3B). Additionally, we stained these EVs using different markers for astrocytes, including aquaporin 4 (AQ4), phosphatidylserine (PS), and mitochondria (DIOC6). Heatmaps show the individual sample percentages (Figure 3C) and MFI (Figure 3E) of the different markers. There were no significant differences between the groups in the percentages of EVs positive for AQ4, PS or DIOC6 or in the MFI of AQ4, PS, or DIOC6 (Figures 3C–F), although a trend of increased AQ4 was seen in 3xTg-AD A-EVs. Therefore, the increased number of A-EVS from 3xTg-AD astrocytes suggests that EVs could be the factor inducing NVU damage in the CM.

### Adult 3xTg-AD A-EVs Induced Neurotoxicity and Astrocyte Hyperreactivity

**FIGURE 4:**
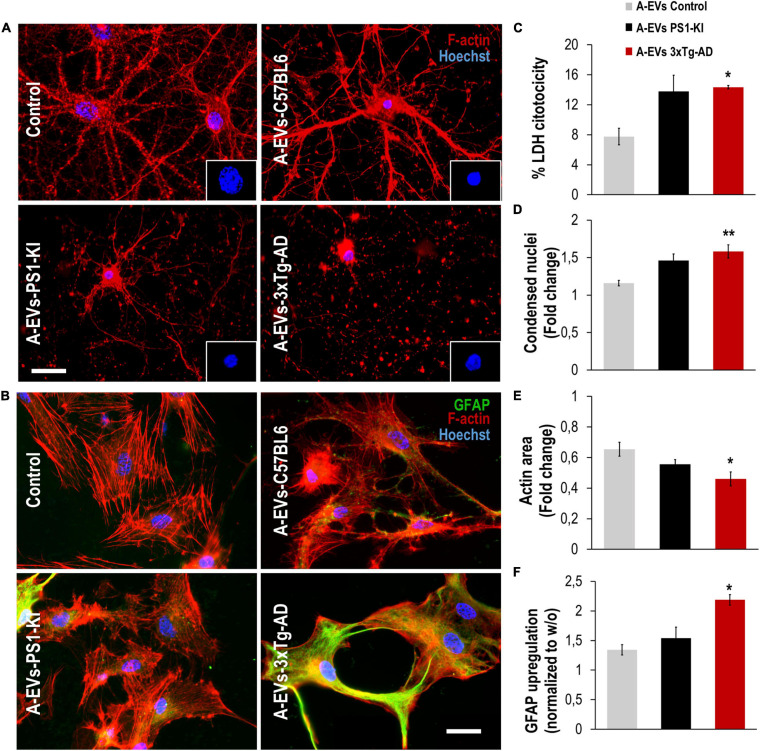
3xTg-AD A-EVs induce astrocyte-neuronal damage. **(A)** Morphological characterization showing neurons: F-actin in red and nuclei were stained with Hoechst (blue). Magnification × 60, scale bar 20 μm. **(B)** The cytotoxicity of co-culture astrocytes-neurons is expressed as the percentage of LDH released from cells 24 h after C57BL6-, PS1-KI and 3xTg-AD A-EVs. **(C,D)** Fold change data calculated by dividing the values by the value obtained from cells with no A-EVs treatment. **(C)** fold change of the percentages of F-actin per field of neurons and **(D)** fold change of the percentage of condensed nuclei of neurons treated with C57BL6-, PS1-KI and 3xTg-AD A-EVs. **(E)** Morphological characterization showing astrocytes: F-actin in red, GFAP in green and nuclei in blue. Magnification × 60, calibration bar 20 μm. **(F)** Fold change data calculated by dividing the values by the value obtained from cells with no EV treatment, Fold change of GFAP intensity (MFI) of astrocyte. Representative data from *n* = 3. Data were plotted as means and SEM. Kruskal–Wallis test and Dunn’s multiple comparison test. ^∗^Indicates *p* < 0.05; ^∗∗^ indicates *p* < 0.01.

To assess whether EVs could be the factors found in CM that are associated with its detrimental effects on the NVU, we treated neuron-astrocyte murine cocultures with C57BL6, PS1-KI and 3xTg-AD A-EVs (Figures 4A,E). C57BL6 and PS1-KI A-EVs induced cytotoxicity (approximately 5–15% of maximum LDH release), while 3xTg-AD A-EVs induced a significant increase in cytotoxicity (Figure 4B) compared with control A-EVs. Additionally, 3xTg-AD A-EVs reduced the F-actin area, increased the number of condensed neuronal nuclei (Figures 4C,D) and increased the MFI of GFAP in astrocytes compared to C57BL6 A-EVs (Figure 4F). These results suggest that 3xTg-AD A-EVs found in CM have a detrimental effect on NVU components.

### Human AD Astrocytes Shed Aquaporin-Positive EVs

**FIGURE 5:**
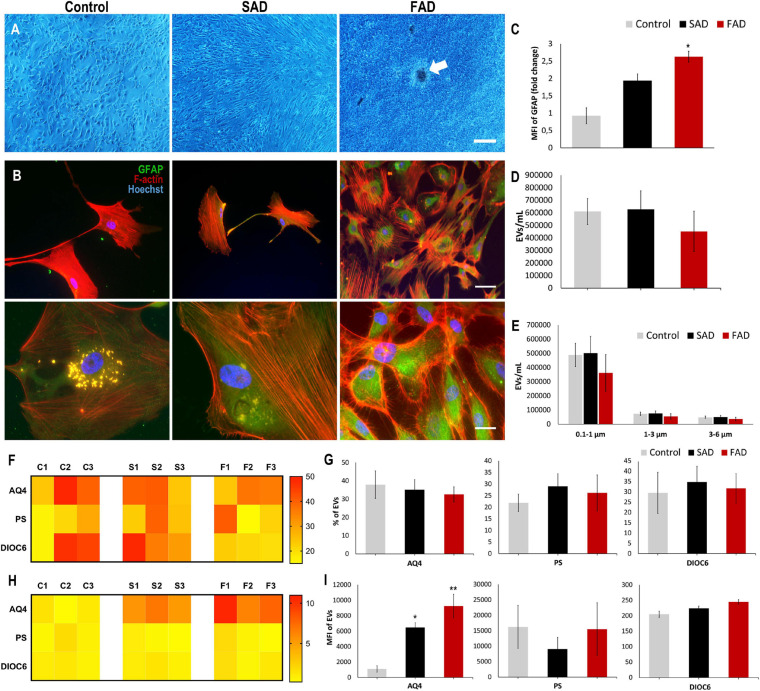
Postmortem Human CNT, SAD and FAD astrocytes in culture and characterization of AD A-EVs. **(A)** Representative images show astrocyte cultures after 50 days. Magnification × 4, scale bar 40 μm. White arrow indicates focal cell aggregates in FAD culture. **(B)** Morphological characterization showing the following: F-actin visualized in red, GFAP visualized in green and nuclei in blue. Magnification × 20, scale bar 100 μm, magnification × 60, scale bar 10 μm. **(C)** MFI of GFAP data seen in B. 20X Representative data from CNT, *n* = 2; SAD, *n* = 3; FAD, *n* = 3. **(D)** Concentration of EVs/mL measured by flow cytometry. **(E)** Concentration of EVs/mL measured In the different size intervals: MVs, 0,1-1 μm; Apoptotic bodies, 1-6 μm. **(F)** Heatmap of percentages of EVs positive for cell markers (AQ4, astrocyte; Annexin, phosphatidylserine (PS) and DIOC6, mitochondria) from each CNT, FAD, and SAD. **(G)** Comparative of percentages of EVs positive of cell markers for grouped data seen in panel **(F)**. **(H)** Heatmap of the normalized MFI of cell markers (to mean data from CNT individuals). **(I)** Comparative of MFI of cell markers for grouped data seen in panel **(H)**. Representative data from CNT, *n* = 3; SAD, *n* = 3; FAD, *n* = 3. Data was plotted as means and SEM. One-way ANOVA, Tukey’s multiple comparison test. ^∗^Indicates *p* < 0.05; ^∗∗^ indicates *p* < 0.01.

In a similar manner, we evaluated astrocytes and A-EVs from *postmortem* sporadic (SAD) and familial AD (FAD) patients and healthy individuals (CNTs). We obtained astrocyte cultures from frontal cortex brain samples at a maximum of 5 h after death (Table 1). After 50 days in culture, we observed 90–100% confluent cultures (Figure 5A). Interestingly, FAD cultures exhibited higher confluence and focal cell aggregates (Figure 5A). We stained these cells for the specific astrocytic markers GFAP and glutamine synthetase (GS). We found that all isolated cells were GFAP + (Figure 5B) and GS + (data not shown). Additionally, a higher MFI of GFAP was found in SAD and FAD cells than in CNT cells (Figure 5C). Astrocytes release EVs in culture conditions (You et al., 2020). Therefore, we compared EVs shed by astrocytes from healthy individuals (CNTs) and patients with FAD or SAD under the same culture conditions. A-EVs from the different study groups showed a similar concentration and size distribution (Figures 5D,E). Additionally, we stained these EVs using different markers for astrocytes (AQ4), phosphatidylserine (PS) and mitochondria (DIOC6). A-EVs did not show differences among the groups in the percentages of EVs that were positive for AQ4, PS or DIOC6 (Figures 5F,G) or in the MFI of AQ4, PS or DIOC6 (Figures 5H,I). Remarkably, we observed a significantly higher expression of AQ4 in FAD and SAD A-EVs than in CNT A-EVs (Figure 5I). These findings show that AD astrocytes exhibit hyperreactivity and release A-EVs with increased aquaporin 4 levels.

### FAD A-EVs Induced Neurotoxicity and Astrocyte Hyperreactivity

**FIGURE 6:**
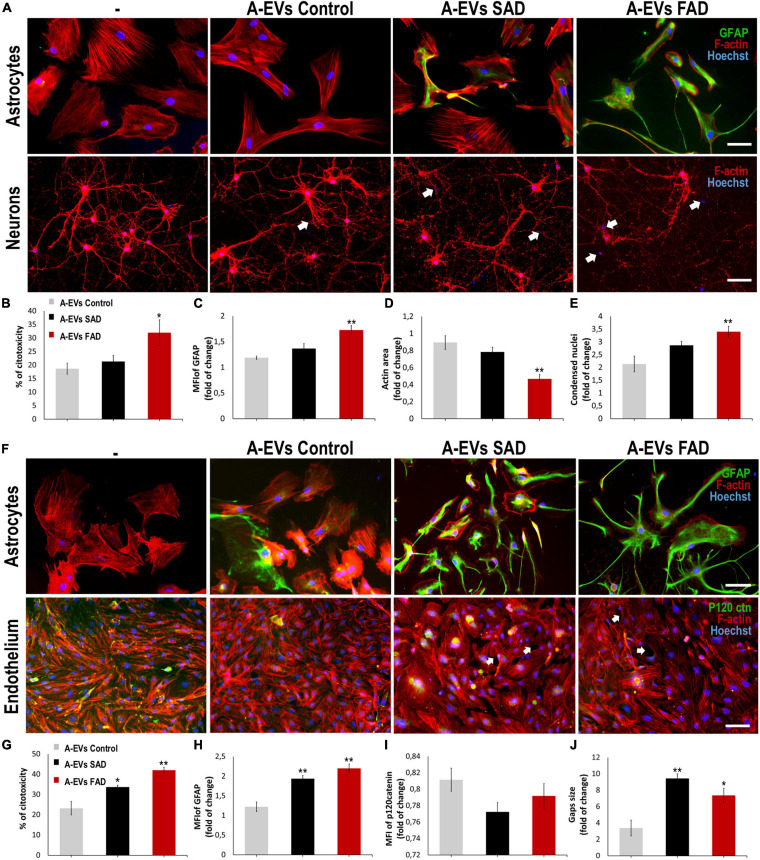
AD A-EVs induce neuroglial and astrocyte-endothelial damage. **(A)** Morphological characterization showing the following: Astrocytes: F-actin in red, GFAP in green and nuclei in blue. Magnification × 20, scale bar 100 μm. Neurons: F-actin in red and nuclei were stained with Hoechst (blue). Magnification × 20, scale bar 100 μm. White arrows indicated condensed nuclei in neurons. **(B)** The cytotoxicity of co-culture astrocytes-neurons is expressed as the percentage of LDH released from cells 24 h after CNT-, SAD-, FAD A-EVs. (C-D) Fold change data calculated by dividing the values by the value obtained from cells with no EV treatment. **(C)** Fold change of GFAP intensity (MFI) of astrocyte, **(D)** fold change of the percentages of F-actin per field of neurons, and **(E)** fold change of the percentage of condensed nuclei of neurons treated with CNT-, SAD-, FAD A-EVs. Representative data from CNT, *n* = 3; SAD, *n* = 3; FAD, *n* = 3. Data was plotted as means and SEM. One-way ANOVA, Tukey’s multiple comparison test. ^∗^Indicates *p* < 0.05; ^∗∗^ indicates *p* < 0.01. **(F)** Morphological characterization of astrocytes: F-actin in red, GFAP cytoskeleton in green and nuclei in blue. Magnification × 20, scale bar 100 μm. Endothelia: F-actin in red, p120catenin in green and nuclei in blue. Magnification × 20, scale bar 100 μm. White arrows indicate gaps in endothelial cells. **(G)** The cytotoxicity of astrocytes-endothelial co-culture expressed as the percentage of LDH released from cells 24 h after CNT-, SAD-, FAD A-EVs. **(H)** Fold change of GFAP and **(I)** p120 catenin intensity (MFI) of astrocytes and endothelia treated with CNT-, SAD-, FAD A-EVs, respectively. **(J)** Fold change of gap size of endothelia treated with CNT-, SAD-, FAD-EVs. CNT, *n* = 3; SAD, *n* = 3; FAD, *n* = 3. Data was plotted as means and SEM. One-way ANOVA, Tukey’s multiple comparison test. ^∗^Indicates *p* < 0.05; ^∗∗^ indicates *p* < 0.01.

A-EVs regulate brain homoeostasis (Hayakawa et al., 2016) and disease response (Dickens et al., 2017; You et al., 2020). To assess the effect of AD A-EVs on the NVU, we first treated neuron-astrocyte murine cocultures (Figure 6A). CNT and SAD A-EVs induced cytotoxicity (approximately 20% LDH release), reduced the F-actin area and increased condensed nuclei in neurons (Figures 6B,D,E). SAD A-EVs also increased the MFI of GFAP in astrocytes compared to CNT A-EVs (Figure 6C), while FAD A-EVs induced higher neurotoxicity and astrocyte hyperactivation (Figures 6B,C). Remarkably, FAD A-EVs induced significantly higher cytotoxicity (approximately 30% of maximum LDH release) than CNT A-EVs (Figure 6B). Additionally, FAD A-EVs induced F-actin redistribution (Figure 6D) and increased GFAP expression and stellate-like morphology in astrocytes compared to CNT A-EVs (Figure 6B). In neurons, FAD A-EVs induced neurotoxicity, as shown by decreased neuronal branching, decreased F-actin area and increased condensed nuclei compared to those in neurons treated with CNT A-EVs (Figures 6A,D,E). These results suggest that AD A-EVs have a detrimental effect on neuroglial components, and this effect is enhanced in cells treated with FAD A-EVs.

### Human AD A-EVs Induce Endothelial Disruption and Astrocyte Hyperreactivity

Because astrocytes regulate BBB homeostasis (Hawkins and Davis, 2005), we evaluated the effects of FAD, SAD and CNT A-EVs in endothelium-astrocyte murine coculture. CNT A-EVs induced cytotoxicity (approximately 20% of maximum LDH release), but SAD and FAD A-EVs induced significantly higher LDH release in these cocultures than CNT A-EVs (Figure 6G). To assess the cellular effects induced by EVs in cocultures, we analyzed astrocytic and endothelial markers (Figure 6F). Both FAD and SAD A-EVs induced astrocyte hyperreactivity (increased GFAP) and endothelial disruption compared to CNT A-EVs (Figures 6F,H). Additionally, both SAD and FAD A-EVs induced astrocyte stellation associated with F-actin redistribution (Figure 6F). Although CNT A-EVs slightly decreased the level of the adherent junction protein p120 catenin (Figure 6I) and increased the gap size between endothelial cells, both SAD and FAD A-EVs induced a significant increase in gap area compared to CNT A-EVs (Figure 6J). Interestingly, SAD A-EVs induced decreased the MFI of the adherent junction protein p120 catenin and the cytosolic-nuclear distribution of this molecule in endothelial cells (Figures 6F,I). These results suggest that both types of AD A-EVs induce endothelial disruption and astrocyte hyperreactivity along the BBB.

### Human AD A-EVs Induced GS Astrocyte Alteration and Higher Vessel Diameter *in vivo*

**FIGURE 7:**
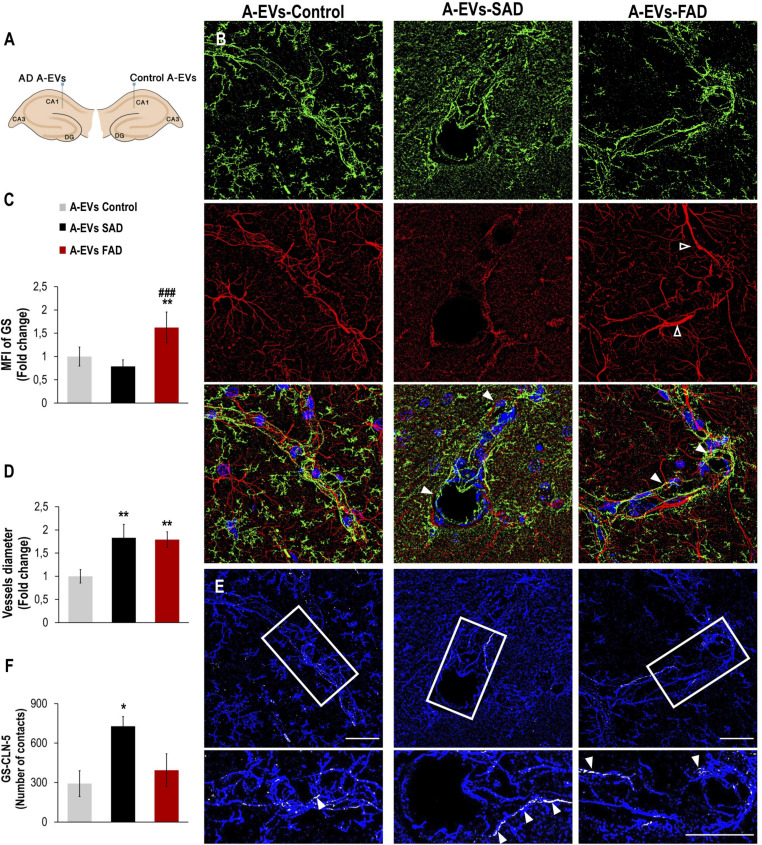
Alzheimer’s disease EVs but not control EVs injection cause GS astrocyte alteration and higher vessel diameter in wild-type mouse brains. **(A)** A schema illustrating EVs from human A-EVs bilaterally injected to the hippocampus of C57BL6 mice at 10 months of age. **(B)** Representative of maximal Z-projection of Hoescht (blue), CLN-5 (green), GS (red) staining 48 h after intrahippocampal injection of CNT, FAD- and SAD A-EVs into aged C57BL6 mouse brain. Original magnification: × 60 (zoom 1.6). White arrowheads indicate regions where high vascular diameter, while empty arrowheads point to increased GS. **(C)** Quantification of the Z projected intensity of GS. ANOVA comparations of A-EVs FAD with ^∗^ A-EVs Control and ^#^ A-EVs SAD, levels of significance were set to *p < 0.05, **p < 0.01, and ^###^*p* < 0.001. **(D)** Quantification of vessels diameter using of the CLN-5 Z projected images. ^∗^Comparison with AEVs Control, levels of significance were set to **p < 0.01. **(E)** Segmented images of GS endfeet (white) contacting CLN-5 vessel (blue). White arrowheads indicate regions where focal GS vascular coincides with CLN-5 staining. The insets are crop magnifications of double positive GFAP-CLN-5 areas (white). **(F)** Quantification of the endfeet volume of double positive GFAP-CLN-5. ^∗^Comparison with A-EVs Control, levels of significance were set to *p < 0.05. Scale bar: **(B,E)**, 20 μm. Data are presented as mean ± SEM from mice treated with CNT- (*n* = 3), SAD- (*n* = 3), and FAD- (*n* = 3) A-EVs; independent experiments.

While EVs derived from AD brains and neurons have been found to induce Tau phosphorylation *in vivo* (Aulston et al., 2019; Ruan et al., 2020), the neurovascular consequences of AD A-EVs *in vivo* remain unclear. To investigate this, EVs isolated from *postmortem* CNT and SAD or FAD astrocytes were unilaterally injected into the lateral and ipsilateral CA1 (±0.80, −1.94, and −1.5) hippocampi, respectively, of wild-type mice (Figure 7A). Staining of an astrocytic marker (GS) and an endothelial marker (CLN-5) in the CA1 region of mice was analyzed at 48 h postinjection. We analyzed the CA1 vasculature located 30-90 microns from the injection point in both hemispheres (Figure 7A). Therefore, the mouse ipsilateral hemisphere, which was treated with FAD A-EVs, displayed increased GS reactivity (Figures 7A–C) at multiple sites compared to the mouse lateral hemisphere, which was treated with control A-EVs. Although both AD A-EVs increased vessel diameter, SAD A-EVs significantly damaged endothelial cells and astrocytes. In response to SAD A-EVs, blood vessels showed strong irregularity and aberrations compared to control blood vessels (Figures 7A–D). Going in depth, the GS and CLN-5 images were segmented and analyzed to determine the possible interaction of GS-reactive astrocytes along the CLN-5 microvasculature (Figures 7D,E). We found that SAD A-EVs increased the number of GS contacts on the CLN-5^+^ vessels and resulted in a continuous pattern of CLN-5 throughout the vessel (Figures 7E,F). These results suggest that AD A-EVs induce gliovascular alterations associated with astrocyte hyperreactivity and endothelial disruption, and this effect is enhanced in cells treated with SAD A-EVs.

### Perivascular Reactive Astrocytes Are Associated With Vascular Deterioration in Human AD

**FIGURE 8:**
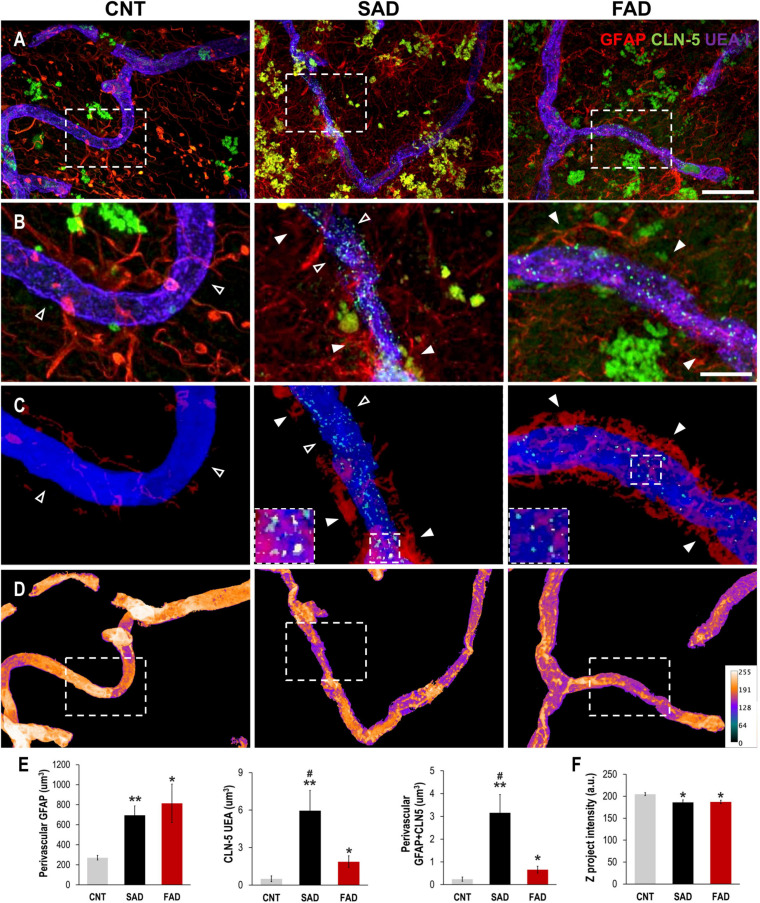
Perivascular GFAP processes are related to altered CLN-5 expression and structural deterioration of the brain vessels in human AD. **(A)** Representative images of triple immunolabeling of GFAP, CLN-5 and the endothelial marker UEA I showing increased astrocytic reactivity near the vessels, and augmented CLN-5 expression in SAD and FAD. **(B)** 2X magnifications of the insets in panel **(A)** highlighting the augment of both perivascular GFAP processes and vascular CLN-5 in AD. **(C)** Segmented images of GFAP processes present between 0 and 2 μm away from the vessel surface showing the proximity of CLN-5 with vascular areas covered by GFAP. White arrowheads indicate regions where high GFAP vascular coverage coincides with high CLN-5 staining, while empty arrowheads point to areas where both GFAP and CLN-5 are scarce. The insets are 2X magnifications of double positive GFAP-CLN-5 areas. **(D)** Heat map representation of superposed UEA I signals indicating structural alteration as intensity values go down. Note that the boxes in panel **(D)** that are enclosing vascular regions with low intensity values, correspond to the images shown in panel **(C)** which display increased GFAP and CLN-5. **(E)** Quantification of the perivascular volume of GFAP, vascular CLN-5, and perivascular double positive GFAP-CLN-5. ^∗^Comparison with CNT, levels of significance were set to *p < 0.05 and **p < 0.01. ^#^Comparison SAD with FAD, levels of significance were set to ^#^*p* < 0.05. **(F)** Quantification of the Z projected intensity of UEA I. ^∗^Comparison with CNT, levels of significance were set to *p < 0.05. Scale bar: **(A,D)**, 20 μm; **(B,C)**, 10 μm. Data are presented as mean ± SEM from CNT *n* = 5, SAD *n* = 4, and FAD *n* = 7; independent experiments.

To evaluate whether the endothelial alterations associated with astrocytes and A-EVs *in vitro* and *in vivo* could be reproduced in the context of human AD, we analyzed the association of brain vessels with astrocytes and their structural integrity in the human frontal cortex of CNTs, SADs and FADs. The GFAP signal increased in the vicinity of the blood vessels in both types of human AD (Figures 8A,B). Similarly, CLN-5 appeared to be increased on the surface of the vessels of both the SAD and FAD samples (Figure 8B). To confirm the extent of perivascular astrocytic process changes between AD and CNTs, GFAP signals located more than 2 μm away from the vessel surface were filtered out (Figure 8C). Thus, we defined GFAP present between 0 and 2 μm away from the vessel surface as astrocytic perivascular processes. This analysis showed that vascular coverage by astrocytic processes was greater in both the SAD and FAD samples (CNT = 271.2, SAD = 694.8, and FAD = 813.9 μm^3^; Figure 8E). Likewise, CLN-5 is increased at the surface of the brain vessels of SAD and FAD samples compared to CNT samples, and SAD exhibited the higher CLN-5 volume between the two AD conditions (CNT = 0.50, SAD = 5.95, and FAD = 1.87 μm^3^; Figure 8E). Moreover, interestingly, most of the CLN-5-positive regions were associated with portions of the vessels that were enriched in GFAP-reactive perivascular processes. Thus, the volume of CLN-5-GFAP double-staining was significantly higher in SAD samples than in CNT and FAD samples, while in FAD samples, the volume of CLN-5-GFAP double-staining was double that in CNT samples (CNT = 0.24, SAD = 3.15, and FAD = 0.66 μm^3^; Figure 8E). Furthermore, we evaluated the brain vessels at the structural level by analyzing the intensity of the superposed UEA I signal. As shown in Figure 8D, UEA I staining appeared more irregular and less intense through the vascular surface in AD. Thus, the intensity of blood vessels was markedly lower in the SAD and FAD samples than in the CNT samples (CNT = 204.8, SAD = 186.1, and FAD = 187.1 a.u.; Figure 8F). Interestingly, both CLN-5- and GFAP-reactive perivascular processes were also frequently found close to the portions of the vessels exhibiting lower intensities. Additionally, other structural parameters of the brain vasculature, such as the fold change of gap area and number of structures, revealed compromised integrity of the blood vessels in AD samples, especially in the SAD samples (Supplementary Figure 3). Taken together, these results showed that structural impairment and altered expression of CLN-5 in the vasculature in the frontal cortex of the human AD brain are associated with astrocytic GFAP-reactive perivascular processes.

## Discussion

In the present study, we determined that murine 3xTg-AD and human AD astrocytes impair neurovascular cells, which we propose could be mediated by EVs. Therefore, we present a novel study showing that isolated murine and human A-EVs from 3xTg-AD mice and SAD and FAD patients affected NVU cell components. We found that EV release was increased in 3xTg-AD astrocytes and that increased AQ4-positive A-EVs from both FAD and SAD samples may be key factors in the alterations of NVU components. Interestingly, A-EVs from 3xTg-AD mice (a mutant model of familial AD) and FAD patients induced cell death and hyperreactivity in neuron-astrocyte cocultures, while A-EVs from both SAD and FAD induced cell death and hyperactivation in endothelium-astrocyte cocultures. SAD and FAD A-EVs induced astrocyte hyperreactivity and vascular deterioration *in vivo*. We associated this finding with perivascular hyperreactive astrocytes and vascular deterioration in the human AD brain. These results indicate that A-EVs damage components of the NVU, which can contribute to the understanding of the disease and could be a source of future interventions.

It was found that while PS1-KI mice are informative for studying the effects of mutations in presenilin-1 (PS1) on the cellular components of the NVU, the mutation exhibited by this model is different from the mutation exhibited by the human FAD astrocytes used in this study. Thus, PS1-KI CM affected the neurovascular components evaluated in this study, but the effect could not be verified by isolating EVs from CM. On the other hand, both CM and isolated EVs from 3xTg-AD astrocytes induced alterations in the cellular components of the NVU, an effect that was validated with EVs from FAD and SAD human astrocytes. However, although the effect was replicated in AD mice and human AD patients, to what extent the AD context of astrocytes from murine models is comparable with the AD context of human astrocytes should be assessed.

Extracellular vesicles are important mediators of NVU function and brain homeostasis (Frühbeis et al., 2013; Pegtel et al., 2014; Otero-Ortega et al., 2017; Prada et al., 2018; Ramirez et al., 2018; Zagrean et al., 2018; Delpech et al., 2019). EVs are released by NVU cells (Pegtel et al., 2014). A-EVs provide trophic support to neurons, mediate vascular homeostasis (Pegtel et al., 2014; Delpech et al., 2019) and cross the BBB where they interact with different organs (Schindler et al., 2014). We observed differences in the concentrations of adult 3xTg-AD A-EVs that were related to 3xTg-AD hyperreactivity. It should be noted that we found alterations in astrocyte function in the neonatal stage, independent of chronic accumulation of Aβ oligomers, which starts after 6 months of age in 3xTg-AD mice, and of Tau, whose accumulation is evident after 15 months of age (Oddo et al., 2003b); however, these are research questions that must be further investigated. Here, we suggest that differences in EVs released from 3xTg-AD astrocytes and AD patient astrocytes may be a key factor in alterations in communication between the NVU components in an AD context. Specifically, we found that these EVs induced neurotoxicity, astrocyte hyperreactivity and vascular damage, which are alterations found in AD (Villar-Vesga et al., 2020). The ability of EVs to transfer a cargo from a donor cell to a recipient cell to induce phenotypic changes in the recipient cell has generated substantial interest in the scientific community (Mathieu et al., 2019). Microglial EVs have been shown to deliver toxic forms of amyloid b and IL-1β and induce neuronal death (Yang et al., 2018), while A-EVs have been shown to contain IL-1β and complement (Bianco et al., 2009; Goetzl et al., 2018). Additionally, A-EVs released in response to IL-1β have a differential proteomic profile and induce differential neuronal uptake, differentiation and firing (You et al., 2020). Accordingly, it would be very interesting to evaluate the effect of AD A-EVs on other NVU cells, such as microglia, and the effect of AD microglia-derived EVs on other components of the NVU. Astrocytes are critical regulators of neuronal function; however, only a few mediators of astrocyte-to-neuron communication have been identified. Astrocytes play a predominant role in the development and maturation of dendritic spines (Haber et al., 2006; Nishida and Okabe, 2007; Perez-Alvarez et al., 2014). In this context, morphological changes generated by AD A-EV treatments can impair the regulation of neuronal excitability. A filopodia-like morphology of mature dendritic spines with a length over 2 μm may lead to hyperexcitability and neuronal death (Yuste and Bonhoeffer, 2001; Kasai et al., 2003; Hotulainen and Hoogenraad, 2010). In this scenario, AD mutations may alter the ability of astrocytes to communicate with other cell types and very likely influence the development and maturation process of dendritic spines and their role in neurotransmission.

Loss of BBB integrity results in increased vascular permeability and is associated with reduced cerebral blood flow and impaired hemodynamic responses (Iadecola, 2004, 2013; Zlokovic, 2011; Kisler et al., 2017). Reduced capillary length, which is associated with endothelial degeneration, reduces the expression of binding proteins, and changes in the capillary basal lamina in the brain tissue of patients with AD have been reported (Bailey et al., 2004; Iadecola, 2004; Baloyannis and Baloyannis, 2012; Sengillo et al., 2013). As explained previously, astrocytes project the “endfeet” of their processes onto the endothelial cells of blood vessels to cover the CNS vascular system (Mathiisen et al., 2010) and regulate perivascular homeostasis, BBB integrity, communication with the peripheral immune system, endothelial transport, and blood supply in response to neuronal activity (Abbott et al., 2010b; Alvarez et al., 2013; Boulay et al., 2016). The finding that 3xtg-AD astrocytes can alter intercellular junctions in the endothelium through A-EVs, potentially independent of Aβ and NFT accumulation, reveals a new perspective in the study of the pathophysiological mechanisms that converge during AD development. Although CM from 3xTg-AD astrocytes and human AD A-EVs were sufficient to induce alterations in endothelial cells, it would be interesting to confirm the effects of CM by isolating EVs and applying them to endothelial cells in culture.

Furthermore, our study compared FAD and SAD AD-EVs under the same culture conditions and without stimuli. In this context, we did not find differences in size or concentration between FAD and SAD AD-EVs; however, we found that AQ4 expression was higher in SAD and FAD A-EVs. AQ4 has been reported to be mislocalized in AD brains (Zeppenfeld et al., 2017), where it is associated with altered waste disposal and microvascular function (Zeppenfeld et al., 2017; Valenza et al., 2019). Interestingly, we observed an increase in AQ4 expression in both SAD and FAD EVs that was associated with vascular effects. A neurodegenerative context in the brain parenchyma modulates the cell state (De Luca et al., 2018), which could change the content of shed EVs. Inflammatory glia can transfer miRNA via EVs to neurons, inducing synaptic alterations (Prada et al., 2018), which could be transferred in A-EVs from AD patients. We hypothesize that the protein, lipid and genetic content of A-EVs may be altered. This content may include proinflammatory cytokines, proteins involved in lipid metabolism (ApoE), proteases, neurotransmitters, protein aggregates, toxic proteins (p-tau and amyloid-β), chaperones, integrins and other functional proteins (Goetzl et al., 2018; Marttinen et al., 2018; Muraoka et al., 2019), as has been previously reported. Proteomic, lipidomic and genetic profiling of these vesicles could be performed because the protein profile in these A-EVs could be associated with AD pathophysiology (Delpech et al., 2019).

Recent *in vivo* research demonstrated that EVs released by neurons that harbor FAD mutations were sufficient to induce tau hyperphosphorylation in healthy brain tissue (Aulston et al., 2019). Using a similar approach, we showed for the first time that AD A-EVs induced gliovascular disturbance; these effects were different between FAD and SAD A-EVs. Specifically, SAD A-EVs damaged the greater vascular structure, which was associated with astrocytic GS loss, while FAD A-EVs affected vascular cells to a lesser extent and promoted increased GS reactivity. The latter effect could be associated with altered astrocytic glutamate function (Hakvoort et al., 2017). The decrease in GS in CA1 cells treated with SAD A-EVs might be related to insufficient glutamine synthetase activity during synaptogenesis, which triggers spatial memory impairment in adult mice, and an age-dependent decrease in glutamine synthetase expression in hippocampal astroglia in an AD context (Olabarria et al., 2011; Son et al., 2019).

Through intimate association with the cerebral circulatory system, astrocytes influence the integrity of the blood vessels and support their functional properties. Accordingly, alteration of astrocytic vascular coverage has been postulated as a key mechanism of pathology (Muoio et al., 2014). Here, we show that increased perivascular GFAP levels are frequently accompanied by higher CLN-5 levels and structural alterations of vessels in the human frontal cortex in AD. CLN-5 is crucial to the integrity and selective permeability of the BBB (Lv et al., 2018), and astrocytes regulate its expression through the release of soluble factors (Obermeier et al., 2013; Zhao et al., 2015). For instance, reactive astrocytes alter tight junctions and promote BBB breakdown by increasing matrix metallopeptidase 9 (MMP-9) release (Renner et al., 2013; Sweeney et al., 2018). Although the loss of vascular CLN-5 has typically been reported in brain pathologies (Biron et al., 2011), previous studies have also shown that it can be redistributed and support peripheral leucocyte infiltration during neuroinflammation (Paul et al., 2016; Stankovic et al., 2016) and that CLN-5-positive extracellular vesicles potentially increase near the surface of brain vessels in human AD (Villar-Vesga et al., 2020). In any case, the disturbances in the expression of CLN-5 that we observed suggest deterioration of the barrier properties of the brain vasculature in AD, which could be related to the damage induced by AD A-EVs.

Furthermore, prior studies have noted that in response to hypoxia and chemical stress conditions such as those occurring in AD, the architecture of the cerebral circulatory system can be remodeled (Obermeier et al., 2013; Zhao et al., 2015). As mentioned in the literature, astrocytes can intervene in these structural modifications by releasing TGF-β1 (Diniz et al., 2019) and recruiting endothelial progenitor cells to lesioned areas (Huang et al., 2019), controlling angiogenesis and vascular repair. Thus, considering the *in vitro* and *in vivo* data described above, and given that the increase in perivascular GFAP, the augmentation of vascular CLN-5, and the structural alteration of the vessels were found to be closely related to each other, our results suggest that astrocytes mediate endothelial instability in human AD, and this is likely to be driven by secreted factors and particles such as EVs.

Previously, our investigations showed that vascular damage and astrocyte activation are induced by systemic EVs and A-EVs in SAD (Villar-Vesga et al., 2020). We detected EV-like structures containing CLN-5 in human SAD brains, and we detected endothelial and leukocyte markers in plasma-derived EVs, which we propose are associated with leukocyte infiltration into the brain parenchyma (Villar-Vesga et al., 2020). Taken together, these results relate to systemic effects that could be associated with environmental risk factors and lifestyle in elderly individuals who have sporadic AD (Lane et al., 2018). Interestingly, FAD systemic EVs damage NVU components to a lower degree at the cellular level than SAD systemic EVs (Villar-Vesga et al., 2020). Here, we found in our *in vitro* experiments that FAD A-EVs induced greater damage (damage to both neuroglial and neurovascular components) to the cellular components of the NVU than SAD A-EVs (damaged neurovascular components). Therefore, we propose that FAD pathology could initiate in the brain through an A-EV pathway, while in SAD pathology, systemic EVs and systemic components have a more pivotal role. Thus, further studies should be performed to understand the pathways involved in the effects of these EVs.

However, our *in vitro* model does not mimic the complex integrity and functionality of the NVU; therefore, in this study, we confirmed the structural damage induced by A-EVs in rodent NVU cells *in vivo*. Furthermore, *postmortem* patients have comorbidities that may influence our analysis. Although AD patients had comorbidities related to hypertension, cardiovascular disease and dyslipidemia, which we related to systemic and metabolic disorders, CNTs also had hypertension and depression as the main comorbidities. Additionally, EV isolation based on size exclusion could determine which type and role of EVs are mostly implicated in AD (Théry et al., 2018). Additionally, determining the protein profiles of AD A-EVs (3xtg-AD, FAD and SAD) using LC-MS/MS analysis could identify factors related to the differences in the changes induced by these AD A-EVs.

## Conclusion

In conclusion, our findings allow us to propose that 3xTg-AD mice and human AD astrocytes impair NVU cells (astrocytes, endothelial cells and neurons) and that this damage could be mediated by EVs. While the deleterious effects associated with 3xTg-AD astrocytes were related to an increase in the number of EVs, the effects of human AD astrocytes were related to increased AQ4 levels in A-EVs. Human AD astrocytes shed EVs that can target different components of the NVU, which could be related to differential disease pathophysiology. A-EVs generated by FAD astrocytes damage both parenchymal neuroglial cells and the endothelium, while EVs from SAD astrocytes damage astrocytes and the endothelium. Nevertheless, further studies are needed to detail the molecular profiles and to elucidate the mechanisms of A-EVs in NVU degeneration.

## Significance Statement

This research shows that *in vitro* 3xTg-AD astrocytes showed an increased GFAP reactivity and 3xTg-AD astrocytes from neonatal and adult mice induced cell-to-cell disruption in the brain microvasculature endothelial cells. Similarly, 3xTg-AD astrocytes conditional media (CM) and astrocytes-EVs (A-EVs) induced neuroglial damage. Cultured human *postmortem* astrocytes from Alzheimer disease (AD) increased GFAP reactivity and released EVs with increased Aquaporin 4 expression. These AD A-EVs induced cytotoxicity and GFAP reactivity in astrocytes. Familiar AD (FAD) A-EVs damaged neuroglial and endothelial components, while sporadic AD (SAD) A-EVs mainly damaged endothelial components. Moreover, AD A-EVs increased astrocyte GS-reactivity and vascular deterioration *in vivo*. This finding could be associated to perivascular reactive astrocytes and vascular deterioration in human AD brain. These results suggest that AD A-EVs are associated with alterations in the cellular components of NVU.

## Data Availability Statement

The original contributions presented in the study are included in the article/Supplementary Material, further inquiries can be directed to the corresponding author/s.

## Ethics Statement

The studies involving human participants were reviewed and approved by Record 119, Agosto 2018-Sede de Investigaciones Universitarias, Universidad de Antioquia. Written informed consent was obtained from the individual(s) for the publication of any potentially identifiable images or data included in this article. The animal study was reviewed and approved by Comité de Ética para la Experimentación con Animales de la Universidad de Antioquia (CEEA). Record 110, May 17th 2017.

## Author Contributions

LG-M, JV-V, JH-R, GC-G, and RP-D designed the experiments and analyzed the data. LG-M, JV-V, and RP-D performed *in vitro* experiments and analyses of research. JH-R performed *in vivo* experiments and analyses of research. AV and FL contributed to sampling and diagnosis of human tissue. JH-R performed human tissue experiments and analyses. LG-M, JV-V, JH-R, and RP-D wrote the manuscript. LG-M, JV-V, JH-R, AV, FL, GC-G, and RP-D reviewed and edited the manuscript. All authors contributed to the article and approved the submitted version.

## Conflict of Interest

The authors declare that the research was conducted in the absence of any commercial or financial relationships that could be construed as a potential conflict of interest.

## Supplementary Material

Supplementary Figure 1 Purity of the astrocyte cell cultures. **(A)** To test the purity of astrocytes cell cultures, different type of astrocytes cultures was labeled with microglia’s marker Iba1, neuron’s marker MAP2 and two astrocyte’s markers, GFAP and S100. **(B)** Percentage labeling cells with each specific marker, quantified in five fields (*n* = 3, a hundred cells were at least analyzed per duplicated). The different types of cell cultures (C57BL6, PS1-KI, and 3xTg-AD) effectively corresponded to cultures with most cells positive to astrocyte marker.

Supplementary Figure 2 CM from adult 3xtg-AD astrocytes induced F-actin spots in endothelial cells *in vitro*. **(A)** Endothelial cells treated with CM from astrocytes for 24 h. Hoechst staining of the nuclei is blue. Phalloidin-Alexa 488 staining of F-actin is green. Threshold details of F-actin spots appearing after treatment with CM from PS1-KI and 3xTg-AD astrocytes. Scale bar: 50 μm. **(B)** Treatment with CM from PS1-KI and 3xTgAD induced a significant increase in the number and total area of F-actin spots in endothelial cells compared with that in the untreated control cultures. Ten fields per treatment were analyzed for each *n*. *n* from 4 to 5. Comparison with ^∗^, Control; #, CM-C57BL6. *P-value* significance: ^∗∗∗^0.001; ^∗∗^0.01; ^∗^0.05.

Supplementary Figure 3 Structural deterioration of the blood vessels is greater in SAD. **(A)** Representative images of segmented blood vessels projected in the Z axis showing structural defects such as gaps (indicated by arrowheads) and number of independent structures (numbered; structures in the edges were excluded). **(B)** Fold change over CNT revealing that blood vessels in SAD display much more gap areas (SAD = 772.73, and FAD = 218.86%) and more independent structures (SAD = 368.58, and FAD = 190.84%) than those in CNT and FAD. Noteworthily, FAD tends also to be higher than CNT in both parameters. Scale bar: 20 μm. Data are presented as mean ± SEM from CNT *n* = 5, SAD *n* = 7, and FAD *n* = 4; independent experiments. Levels of significance were set to ^∗^*p* < 0.05 and ^∗∗^*p* < 0.01.

## Abbreviations

3xTg-AD: triple transgenic Alzheimer’s disease mouse
A β: β-amyloid peptide
AD: Alzheimer’s disease
APP: amyloid precursor protein
A-EVs: astrocytes-EVs
BBB: blood brain barrier
CB: cytoskeleton buffer
CBS: sucrose cytoskeleton buffer
CM: conditional media
CNS: central nervous system
CSF: cerebrospinal fluid
DIV: day *in vitro*
EVs: extracellular vesicles
FAD: familial Alzheimer’s disease
FBS: fetal bovine serum
GFAP: glial fibrillary acidic protein
IF: immunofluorescence
ILV: Intraluminal vesicles
MAP-2: microtubule-associated protein-2
MVs: microvesicles
NB: neurobasal culture medium
NFT: neurofibrillary tangles
NVU: neurovascular unit
PBS: saline phosphate buffer
PS1: presenilin 1
PS1-KI: knock-in mouse for presenilin 1
SAD: sporadic Alzheimer’s disease.
