# Supplementary figures and images for: Extracellular Vesicles From 3xTg-AD Mouse and Alzheimer’s Disease Patient Astrocytes Impair Neuroglial and Vascular Components
Source: Front Aging Neurosci. 2021 Feb 19;13:593927. doi: 10.3389/fnagi.2021.593927 (PMC7933224; doi:10.3389/fnagi.2021.593927)

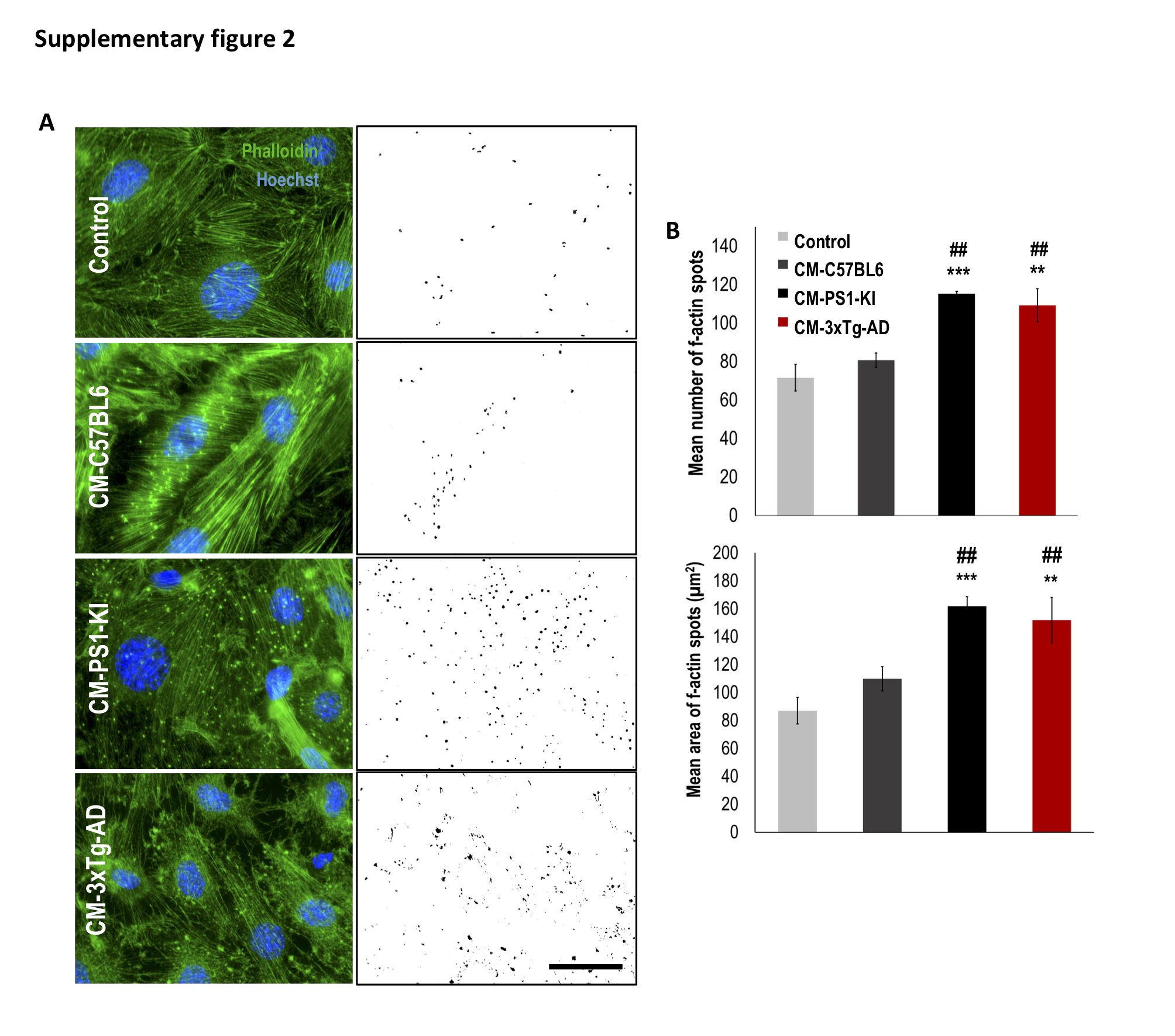

Supplement: Supplementary Figure 2 — CM from adult 3xtg-AD astrocytes induced F-actin spots in endothelial cells in vitro. (A) Endothelial cells treated with CM from astrocytes for 24 h. Hoechst staining of the nuclei is blue. Phalloidin-Alexa 488 staining of F-actin is green. Threshold details of F-actin spots appearing after treatment with CM from PS1-KI and 3xTg-AD astrocytes. Scale bar: 50 μm. (B) Treatment with CM from PS1-KI and 3xTgAD induced a significant increase in the number and total area of F-actin spots in endothelial cells compared with that in the untreated control cultures. Ten fields per treatment were analyzed for each n. n from 4 to 5. Comparison with ∗, Control; #, CM-C57BL6. P-value significance: ∗∗∗0.001; ∗∗0.01; ∗0.05. [file Image_2.TIFF]

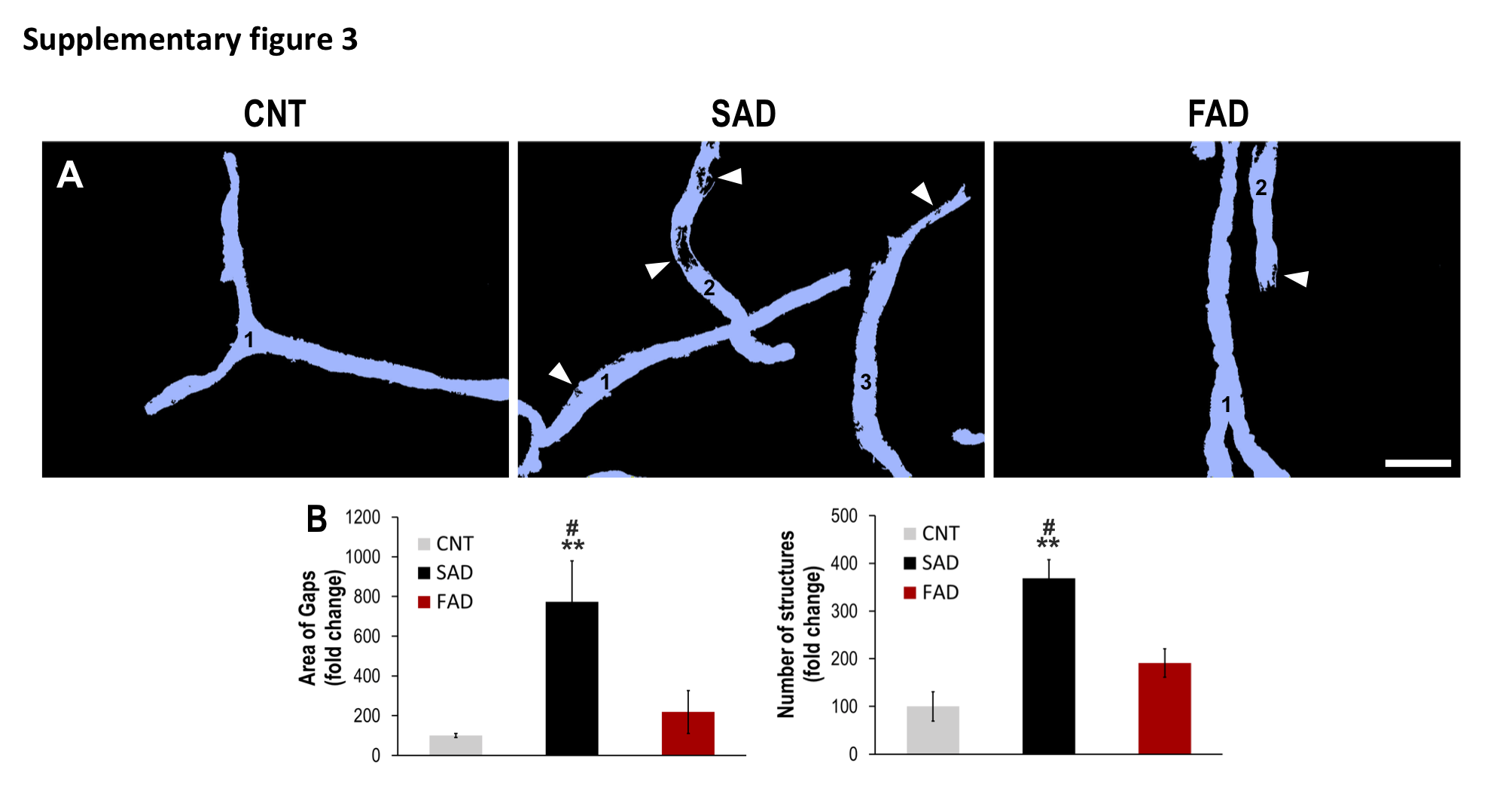

Supplement: Supplementary Figure 3 — Structural deterioration of the blood vessels is greater in SAD. (A) Representative images of segmented blood vessels projected in the Z axis showing structural defects such as gaps (indicated by arrowheads) and number of independent structures (numbered; structures in the edges were excluded). (B) Fold change over CNT revealing that blood vessels in SAD display much more gap areas (SAD = 772.73, and FAD = 218.86%) and more independent structures (SAD = 368.58, and FAD = 190.84%) than those in CNT and FAD. Noteworthily, FAD tends also to be higher than CNT in both parameters. Scale bar: 20 μm. Data are presented as mean ± SEM from CNT n = 5, SAD n = 7, and FAD n = 4; independent experiments. Levels of significance were set to ∗p < 0.05 and ∗∗p < 0.01. [file Image_3.TIFF]
